# Evidence for the Effect of Vaccination on Host-Pathogen Interactions in a Murine Model of Pulmonary Tuberculosis by *Mycobacterium tuberculosis*

**DOI:** 10.3389/fimmu.2020.00930

**Published:** 2020-05-19

**Authors:** Zyanya Lucia Zatarain-Barrón, Octavio Ramos-Espinosa, Brenda Marquina-Castillo, Jorge Barrios-Payán, Fernanda Cornejo-Granados, Otoniel Maya-Lucas, Gamaliel López-Leal, Camilo Molina-Romero, Richard M. Anthony, Adrián Ochoa-Leyva, Inti Alberto De La Rosa-Velázquez, Rosa Gloria Rebollar-Vega, Robin M. Warren, Dulce Adriana Mata-Espinosa, Rogelio Hernández-Pando, Dick van Soolingen

**Affiliations:** ^1^Experimental Pathology Laboratory, Department of Pathology, Instituto Nacional de Ciencias Médicas y Nutrición “Salvador Zubirán”, Mexico City, Mexico; ^2^Departamento de Microbiología Molecular, Instituto de Biotecnología, Universidad Nacional Autónoma de México, Cuernavaca, Mexico; ^3^Department of Genetics and Molecular Biology, Centro de Investigaciones y de Estudios Avanzados (CINVESTAV), Mexico City, Mexico; ^4^Tuberculosis Reference Laboratory, National Institute for Public Health and the Environment (RIVM), Bilthoven, Netherlands; ^5^Genomics Laboratory, Red de Apoyo a la Investigación (RAI), Universidad Nacional Autónoma de México – Instituto Nacional de Ciencias Médicas y Nutrición “Salvador Zubirán”, Mexico City, Mexico; ^6^Division of Molecular Biology and Human Genetics, Department of Biomedical Sciences DST/NRF Centre of Excellence for Biomedical Tuberculosis Research, MRC Centre for Molecular and Cellular Biology, Faculty of Health Sciences, Stellenbosch University, Tygerberg, South Africa

**Keywords:** tuberculosis, BCG vaccination, Beijing genotype, virulence, lung transcriptome

## Abstract

The global control of Tuberculosis remains elusive, and Bacillus Calmette-Guérin (BCG) -the most widely used vaccine in history—has proven insufficient for reversing this epidemic. Several authors have suggested that the mass presence of vaccinated hosts might have affected the *Mycobacterium tuberculosis* (MTB) population structure, and this could in turn be reflected in a prevalence of strains with higher ability to circumvent BCG-induced immunity, such as the recent Beijing genotype. The effect of vaccination on vaccine-escape variants has been well-documented in several bacterial pathogens; however the effect of the interaction between MTB strains and vaccinated hosts has never been previously described. In this study we show for the first time the interaction between MTB Beijing-genotype strains and BCG-vaccinated hosts. Using a well-controlled murine model of progressive pulmonary tuberculosis, we vaccinated BALB/c mice with two different sub-strains of BCG (BCG-Phipps and BCG-Vietnam). Following vaccination, the mice were infected with either one of three selected MTB strains. Strains were selected based on lineage, and included two Beijing-family clinical isolates (strains 46 and 48) and a well-characterized laboratory strain (H37Rv). Two months after infection, mice were euthanized and the bacteria extracted from their lungs. We characterized the genomic composite of the bacteria before and after exposure to vaccinated hosts, and also characterized the local response to the bacteria by sequencing the lung transcriptome in animals during the infection. Results from this study show that the interaction within the lungs of the vaccinated hosts results in the selection of higher-virulence bacteria, specifically for the Beijing genotype strains 46 and 48. After exposure to the BCG-induced immune response, strains 46 and 48 acquire genomic mutations associated with several virulence factors. As a result, the bacteria collected from these vaccinated hosts have an increased ability for immune evasion, as shown in both the host transcriptome and the histopathology studies, and replicates far more efficiently compared to bacteria collected from unvaccinated hosts or to the original-stock strain. Further research is warranted to ascertain the pathways associated with the genomic alterations. However, our results highlight novel host-pathogen interactions induced by exposure of MTB to BCG vaccinated hosts.

## Introduction

In 1921 the Bacillus Calmette-Guérin (BCG) vaccine, constituting of viable but attenuated *Mycobacterium bovis* bacteria, was introduced as the first and so far only tuberculosis (TB) preventive vaccine approved by the World Health Organization (WHO) (1). However, despite billions of individuals having been vaccinated in the past century, TB continues to pose a serious threat to global health. Recently, the WHO reported that TB accounts for ~10 million incident cases, 44% of which are concentrated in the South-East Asian region (2). Furthermore, TB is presently the most deadly infectious disease due to a single pathogen.

The BCG vaccine has been outstandingly successful in preventing severe forms of tuberculosis (meningeal and miliary). Nonetheless, the vaccine presents considerable shortcomings in terms of preventing pulmonary tuberculosis, with a considerable variability in efficacy, ranging from 0 to 75% in different regions of the world (3, 4). Hypotheses to explain this remarkable heterogeneity in efficacy include flaws in the design of long-term studies, genomic differences between the BCG daughter strains, and that BCG protection may differ by *Mycobacterium tuberculosis* (MTB) genotype (5–7). Indeed, the distribution of MTB genotypes differs significantly by geographic area (8), if BCG vaccination protects against some, but not all genotypes to the same extent (9) this may contribute to the success of specific genotypes. If so, there would be an ongoing selection of MTB genotypes with an increased ability to circumvent BCG-induced immunity, particularly in high prevalence countries with high vaccination coverage (5).

The most studied in this respect is the Beijing genotype (recently re-named Lineage 2), highly prevalent in South East Asia, the former Soviet Union, and South Africa (10, 11). The Beijing genotype is part of the modern MTB lineage, and is represented by often genetically highly conserved, widespread strains (12, 13). Moreover, Beijing strains have been significantly associated with the spread of multidrug resistant (MDR)-TB, although recent reports from patients in the Beijing area have challenged previous studies (14–16). Several studies have indicated Beijing strains may have selective advantages over other genotypes; they are more prevalent in young patients, and show high clonality, suggesting ongoing transmission, and are associated with rapid disease progression and unfavorable treatment outcomes (17–19). Moreover, studies in mice have shown that BCG vaccination protects less efficiently against infection by Beijing strains compared with other genotypes, and this impediment to the efficacy of BCG has been linked to a decrease in effector immunity in subjects infected by Beijing-genotype strains (20, 21).

Other factors may also come into play when dissecting the reasons behind the fact that vaccination alone as a prophylactic strategy has not been enough to reverse the TB epidemic. One such factor is the effect of vaccinated hosts on a microorganism's population structure. This effect has been described previously, particularly for “imperfect vaccines,” which act by reducing the growth rate of a microorganism within a host, rather than preventing the infection process and spread to new humans (22). In such cases, higher vaccine coverage would consequentially more efficiently eliminate less virulent forms, driving the selection of microorganisms with a higher degree of virulence. These vaccine-adapted bacteria would possess competitive advantages compared with wild-type bacteria, potentially in both vaccine-naïve and vaccinated individuals (22).

In this study, we hypothesize that exposure to BCG-induced immunity may select vaccine-escape microorganism variants with a higher capacity for disease breakdown. If so, MTB bacilli would increase their virulence, as a function of changes in their genetic makeup, after exposure to BCG-induced immunity in vaccinated hosts. We tested this hypothesis in a syngeneic BALB/c mouse model of progressive pulmonary tuberculosis, by comparing the virulence of two Beijing genotype strains (and control strain H37Rv) before and after passage through BCG-vaccinated and non-vaccinated mice. The course of the disease in terms of pulmonary bacillary loads determined by colony forming units [CFUs]/mL, histopathology assessment of lung tissue and survival curves were used as parameters.

## Methods

### MTB Strain Selection and Culture Conditions

**Figure 1:**
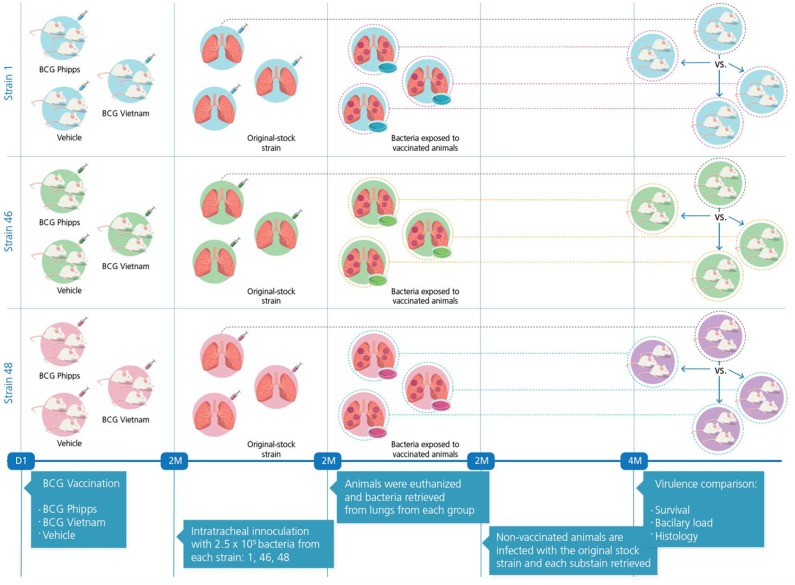
Experimental strategy.

An overview of the experimental design is portrayed in Figure 1. Strains were selected from a collection of clinical isolates from different regions of the world, considering diverse factors for strain selection including genotype and having been previously characterized using a murine model (23) (Table 1).

**Table 1:** Bacterial strains selected for experimentation.

**Code**	**Species**	**Genotype/code**	**Lineage**	**Country of origin**	**Cluster size**
1	*M. tuberculosis*	Euro American	4	United States	n/a (reference strain H37Rv)
46	*M. tuberculosis*	Beijing Typical (2002-1612)	2	Vietnam	4
48	*M. tuberculosis*	Beijing Atypical Sublineage-1 (2697)	2	South Africa	1

Stock bacteria from the selected strains (namely strains 1 [laboratory strain H37Rv; Euro-American lineage], 46 and 48 [Beijing lineage]) were grown in Middlebrook 7H9 broth (Difco Laboratories, Detroit, MI, USA) supplemented with glycerol, Tween-80, and OADC enrichment (containing oleic acid, albumin, dextrose, and catalase) at 35°C, 75 RPM. All cultures were monitored for optic density and obtained at mid log-phase. Bacterial viability was assessed using the LIVE/DEAD BacLight Bacterial Viability Kit (Invitrogen, Eugene, OR) for flow cytometry, following manufacturer's instructions. Briefly, liquid cultures were centrifuged at 10,000 × g for 15 min. The supernatant was removed and the pellet was re-suspended in 2 mL of 0.85% NaCl. One mL of this suspension was added to a new 50 mL tube containing 20 mL of 0.85% NaCl, and incubated at room temperature for 1 h. The sample was centrifuged at 10,000 × g for 15 min and re-suspended in 20 mL of 0.85% NaCl and centrifuged at 10,000 × g in order to remove any traces of growth medium. The pellet was re-suspended in a separate tube with 10 mL of 0.85% NaCl and 1 mL of this suspension was used for assessment of optic density using a spectrophotometer at 600 nm (_OD_600). The suspension was diluted 1:100 in filtered, sterile water and from this stock solution different dilutions were prepared in cytometer-compatible tubes in a total volume of 2 mL. Using a microfuge tube, a mixture was prepared with 35 μL of component A and 35 μL of component B. We added 6 μl of this mixture to each sample and mixed by pipetting. The mixture was incubated in the dark for 15 min at room temperature. Data were acquired using a cytometer using standard procedures. The kit contains both SYTO9 stain and propidium iodide, therefore bacteria are labeled red when dead and green when alive. All MTB strains were required to have >90% live cells in order to be stored and used for animal experimentation. Each strain was plated in serial dilutions in Middlebrook 7H10 agar in order to count the Colony Forming Units (CFUs) per mL and then stored at −80°C in individual aliquots.

### Exposure of Original-Stock Bacteria to the Immune System of BCG-Vaccinated Mice

Groups of five male BALB/c mice 6–8 weeks old were immunized subcutaneously with either one of two different sub strains of BCG (BCG Phipps or BCG Vietnam, 8,000 live bacilli suspended in 50 μl PBS), for a control group, a third group of five mice received a subcutaneous injection of the vehicle solution, Phosphate-Buffered Saline (PBS). Two months post-immunization the mice in each group were challenged with the selected strains (1, 46, and 48) through the intratracheal route. Briefly, each mouse was anesthetized with sevofluorane in a gas chamber, immobilized and inoculated intratracheally using a stainless steel cannula with 0.1 mL of PBS containing 2.5 × 10^5^ live bacteria. Mice were kept in vertical position until they fully recovered. Infected mice were kept in cages fitted with micro-isolators in groups of ≤ six animals per cage. All animal procedures were performed according to the national regulations on animal care and experimentation (NOM 062-ZOO-1999) after approval by the Animal Experimentation Committee at the National Institute of Medical Sciences and Nutrition México (PAT-1860-16/18-1).

Two months after challenge with bacterial strains, mice were euthanized by exsanguination while under anesthesia; lungs were extracted and immediately snap-frozen in liquid nitrogen. Each lung was homogenized in PBS-Tween-80 0.05% and serial dilutions were cultured in Middlebrook 7H10 agar medium at 37°C with 5% CO_2_. Plates were reviewed every week to check for bacilli colony growth. Following 2–3 weeks (2 weeks for H37Rv, 3 weeks for both Beijing strains) after plating, three colonies were placed in 7H9 medium for colony growth and in blood-agar plates in order to ascertain MTB purity. Purity was also assessed at all time points further whenever the bacterial stocks were manipulated by plating a sample in blood-agar, and performing a Ziehl-Neelsen stain and gram stain in order to identify only ziehl-neelsen positive bacteria in all the samples. Any samples in which other microorganisms grew within the blood-agar or were identified using gram staining were disposed of accordingly. A total of three sub strains from each original strain were obtained from vaccinated and control animals: the first one was obtained from mice immunized with BCG Phipps, the second one from mice immunized with BCG Vietnam, and the third one from non-vaccinated, control mice. Growth curves were constructed with each of these sub strains (each assigned a suffix letter P, V, or S in order to reflect whether they had passed through mice vaccinated with BCG-Phipps [P], BCG-Vietnam [V], or Saline-control [S]) and bacilli were collected at the log-phase. All collected bacilli were assessed for viability as previously described. Following, they were seeded in Middlebrook 7H10 agar in serial dilutions in order to count the number of CFUs per mL and stored at −80°C.

### Virulence Assessment

Tubes containing bacteria collected from all experimental groups (P, V, S) as well as the original-stock strain were thawed at 37°C and prepared in order to infect groups of 50 naïve, non-vaccinated, BALB/c mice following the same procedure previously described. Once infected, mice were monitored and then euthanized following the model of progressive pulmonary TB, which has been extensively described elsewhere (24). Briefly, five mice per group were euthanized by exsanguination after total anesthesia at days 1, 3, 7, 14, 21, 28, 60, and 120 following infection. Lungs from euthanized animals were extracted and stored according to their future use: 4 lungs were snap-frozen in liquid nitrogen for future CFU-assessment; 3 lungs were snap-frozen in liquid nitrogen for future RNA extraction; 3 lungs were instilled by the trachea with ethyl alcohol and stored in ethyl alcohol-filled tubes for histopathology evaluation. Methods for these assessments have been extensively reviewed elsewhere (25). Two independent experiments were performed with each set of strains.

#### Evaluation of Lung Bacillary Load Through CFU

The right lung from four of the euthanized animals in each time point was quickly removed after exsanguination and snap-frozen in liquid nitrogen. Lungs were progressively unfrozen and homogenized with a polytron (Kinematica, Lucerne, Switzerland) in tubes containing 1 ml PBS, Tween-80 0.05%. Dilutions of each homogenate were seeded in plates containing 7H10 medium. The plates were placed in incubators at 37°C with 5% CO_2_ for 3 weeks. The number of colonies was counted and extrapolated to show data as number of CFU per mL of lung tissue.

#### Preparation of Lung Tissue for Histopathology Studies

The left lung from three of the euthanized animals in each time point was perfused through the trachea with ethyl alcohol (J:T Baker, Mexico City, México). Lungs were then dehydrated and embedded in paraffin, 4 μm sections were obtained and stained with haematoxylin and eosin in order to evaluate lung pathology. Histopathological parameters evaluated in this study included pneumonia (expressed as percentage of lung area affected); necrosis (expressed as percentage of lung area affected); average number of granulomas per lung (*n* = 3) and average size of granulomas (in μm^2^). Definitions for each parameter have been previously established in this murine model, briefly pneumonia is defined as areas with inflammatory infiltrate that occupied alveolar lumens and alveolar-capillary interstitium. For the quantification of the area of granulomas, all the granulomas [defined as well-delimited nodular aggregates of lymphocytes and macrophages that can include dendritic cells and fibroblasts formed in response to persistent TB infection (24, 26)] were blind measured by a well-trained pathologist in the whole slide at 400X magnification. Necrosis was defined as Areas with total tissue architecture destruction with abundant cellular eosinophilic cytoplasmic debris, along with fragmented or karyorrhectic nuclei. Data is reported as the mean values ± SD from three different subjects at each time-point. All parameters were assessed using an automated image analyzer (QWin Leica, Milton Keynes, Cambridge, UK).

### Statistical Analysis for Virulence Parameters

In order to determine statistical significance in the bacillary load and the histopathology studies we compared groups using a 2-way ANOVA followed by a Bonferroni posttest in order to determine significant differences among the groups (*p* < 0.05 was considered significant). The survival curves were evaluated using a Log-Rank test in order to find differences between the curves along the duration of the study. Each survival analysis was performed with 50 subjects per group, a total of 200 subjects per experimental set of strains (subjects were censured from the survival analysis at the time of euthanasia). All the statistical analysis was performed using GraphPad Prism Software (version 6.0, La Jolla, USA).

### NGS-Sequencing and Bioinformatics

Bacterial gDNA was extracted from liquid cultures and libraries were constructed using the Illumina TruSeq DNA PCR-Free library prep kit (Illumina, San Diego USA, Cat. No. 20015962). Libraries were constructed starting with 2 μg of gDNA and sequenced in paired-end version (2 × 150 bases) using an Illlumina NextSeq500 equipment at the Biotechnology Institute (IBT, Cuernavaca, Mexico). A Beijing-genotype genome NITR203 (RefSeq: NC_021054.1) recently curated by the NCBI was used as a reference strain. Raw sequences were filtered and low quality reads were deleted using Trimmomatic (v 0.33) (27). As previously reported for Beijing-genomes, CLC Genomics Workbench version 10 was used in order to assemble the 4 sequenced strains. Details regarding genome assembly can be found in Supplementary Table 1.

In order to call variants, the Snippy V4.3.6 program was used, with the default setting. Following, the genome was annotated using Prokka (default settings) v1.13 (28). Variants were initially identified between each of the sequenced strains and the reference Beijing genome NITR203. All the variants which were shared between the original-stock strain and the vaccine-exposed strains when comparing with the reference strain were filtered out, and only those variants which were unique to the original-stock strain or to the vaccine-exposed strain were considered biologically relevant for comparison purposes. Unique variants were compared between the original strains and the vaccine-exposed strains which presented a virulence increase (46 vs. 46P, 48 vs. 48V). Further, variants were compared between the two vaccine-exposed strains (46P vs. 48V).

For RNAseq, RNA was extracted from lungs of infected mice at day 21 post-infection and quantified using Qubit; quality was assessed using a Bioanalyzer. Only samples with an RNA integrity number (RIN) >8.0 were used. Libraries were constructed using 1 μg of each RNA sample, using the TruSeq stranded mRNA kit (Illumina, San Diego, USA, 20020594). Libraries were sequenced in single end version (125 bases) in an Illumina HiSeq2500 equipment at the RAI (RAI, Mexico City, Mexico). Raw sequences were trimmed using Trimmomatic (v 0.33) (27). Following, reads were mapped using BWA_MEM (29), against the *Mus musculus* reference genome (GCA_000001635.8 GRCm38.p6). The read count was performed using htseq (30). The differential gene expression (DGE) was assessed using the bioconductor package DESeq2 (version 3.7) (31). The GO (Gene Ontology (GO) enrichment analysis for differentially regulated genes, which considers the functional hierarchy of the differentially expressed genes) and KEGG enrichment were evaluated using WebGestalt (32).

## Results

### Virulence Assessment

#### Survival Rate

**Figure 2:**
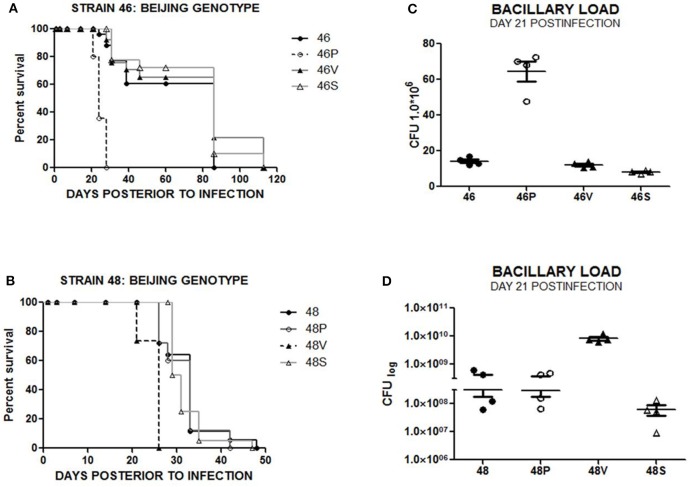
**(A)** Survival rate for animals infected with strains 46, 46P, 46V, and 46 S (*n* = 50 subjects/strain). **(B)** Survival rate for animals infected with strains 48, 48P, 48V, and 48S (*n* = 50 subjects/strain). **(C)** Bacillary load in lungs from animals infected with strains 46, 46P, 46V, and 46S at day 21 postinfection. **(D)** Bacillary load in lungs from animals infected with strains 48, 48P, 48V, and 48S at day 21 postinfection.

In order to test whether immunization exerted a change in the virulence in MTB bacilli exposed to vaccinated mice, groups of 50 naïve BALB/c mice were intratracheally infected with each of the following bacteria: original stock strain (*n* = 50), “P” strain (*n* = 50) isolated from the lungs of BCG Phipps vaccinated mice, “V” strain (*n* = 50) isolated from the lungs of BCG Vietnam vaccinated mice and control “S” strain (*n* = 50) isolated from the lungs of sham-vaccinated, control mice. Survival rates for animals infected with strain 1 (reference strain H37Rv) and vaccine-exposed variants (1P, 1V, and 1S) showed no significant differences (Supplementary Figure 1A). In contrast, animals infected by vaccine immunity-exposed Beijing genotype strain 46P had a significantly decreased survival compared with the original-stock strain 46 (*p* < 0.0001) (Figure 2A). Similarly, in the case of Beijing genotype strains 48, 48P, 48V, and 48S, animals infected with vaccine-exposed strain 48V had a significantly decreased survival when compared with the original-stock strain 48 (*p* < 0.0001) (Figure 2B).

#### Pulmonary Bacillary Load

To further assess the virulence level of the original-stock strains in comparison with the vaccine-exposed strains, the presence of colony forming units (CFU) were compared in lungs from animals infected by original-stock bacilli and vaccine-exposed bacilli. Lungs from animals infected with Euro-American genotype laboratory strain 1 (H37Rv) and vaccine-exposed strains 1P, 1V, and 1S showed no significant difference in terms of bacillary load on days 1, 3, 7, 21, 28, 60, and 120 post-infection (Supplementary Figure 1B), although we observed a four times lower bacillary load in lungs collected from animals infected by strain 1 (4.5 × 10^6^ CFU/mL lung homogenate) compared with those infected by strain 1V (17.4 × 10^6^ CFU/mL lung homogenate) on day 14 post-infection (*p* < 0.01). This difference did not correlate with survival or histological damage (Supplementary Figure 1C). However, in the case of Beijing strains 46, 46P, 46V, and 46S, we observed a significant difference in bacillary load at day 21 post infection when comparing lungs obtained from animals infected by the original strain 46 (1.4 × 10^7^ CFU/mL lung homogenate) and those infected with strain 46P (6.5 × 10^7^ CFU/mL lung homogenate) at day 21 post-infection (*p* < 0.001) (Figure 2C). Similarly, Beijing-genotype strain 48 had a significantly lower bacillary load (3.1 × 10^8^ CFU/mL lung homogenate) compared with vaccine-exposed strain 48V (8.3 × 10^9^ CFU/mL lung homogenate) at day 21 post-infection (*p* < 0.01) (Figure 2D).

#### Histopathological Damage

The third virulence parameter evaluated was histopathological damage. Animals infected by strain H37Rv (Euro-American lineage) strain 1 and vaccine-exposed 1P, 1V, and 1S had pneumonia-type damage, which appeared at day 21 post-infection and increased throughout days 28, 60, and 120 post-infection. A significant difference in lung area affected by pneumonia was observed on day 120 between the original-stock strain 1 (52.6%) and the vaccine-exposed strain 1P (78.6%) (*p* < 0.05); nonetheless, this difference did not correlate with other parameters for evaluating virulence (Supplementary Figure 1C).

**Figure 3:**
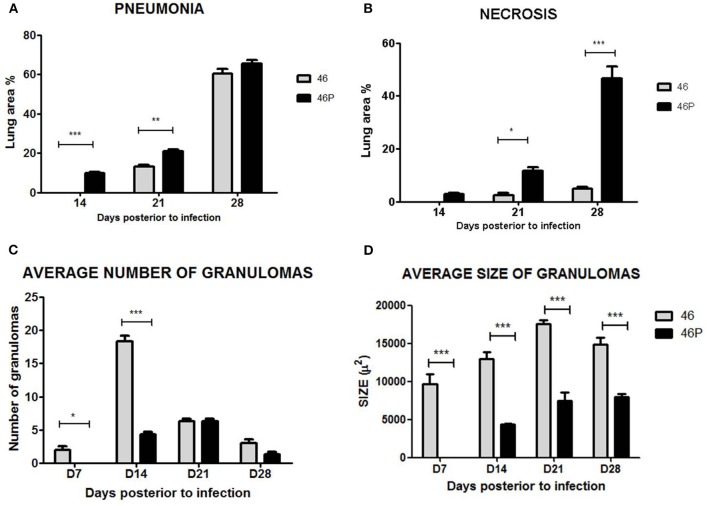
**(A)** Lung area affected by pneumonia in animals infected by strain 46 and 46P. **(B)** Lung area affected by necrosis in animals infected by strain 46 and 46P. **(C)** Average number of granulomas in animals infected by strain 46 and 46P. **(D)** Average size of granulomas in animals infected by strains 46 and 46P. *p < 0.05, **p < 0.01, ***p < 0.001.

**Figure 4:**
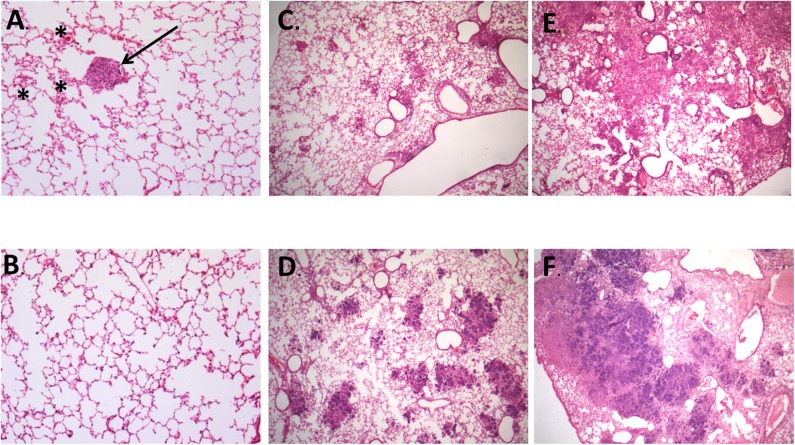
Representative images of histopathological findings in animals infected by strain 46 and strain 46P. **(A)** Animals infected by strain 46 had early formation of granulomas (arrow) and interstitial inflammation, since day 7 post-infection (100X magnification). **(B)** Representative image of animals infected by strain 46P at day 7 postinfection, without formation of granulomas and very scarce if any interstitial inflammation (100X magnification). **(C)** On day 21 postinfection, animals infected by strain 46 had small pneumonic areas throughout the lung tissue (25X magnification). **(D)** Animals infected by strain 46P, instead, had larger pneumonic and necrotic areas by day 21 postinfection (25X magnification). **(E)** By day 28 postinfection, animals infected by strain 46 had larger, confluent pneumonic areas, with scarce areas with necrosis (25X magnification). **(F)** Contrary, animals infected by strain 46P had large areas of massive pulmonary necrosis which occupied most of the lung tissue (25X magnification). Stained with hematoxylin and eosin. * areas of interstitial inflammation.

**Figure 5:**
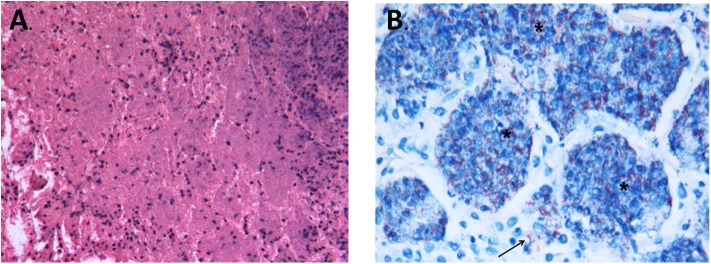
**(A)** Representative image of pulmonary necrosis in animals infected by strain 46P on day 28 postinfection. The image shows the complete loss of pulmonary tissue architecture over large areas, with cellular debris, picnotic and fragmented nuclei and acidophilic material occupying the extracellular space (200X magnification). **(B)** Ziehl-Neelsen stain showing specific necrotic areas with the presence of large ammounts of acid-fast bacteria within the necrotic tissue (*) but also free in the interstitial space (arrow) (1000X magnification; stained with Ziehl-Neelsen).

In correlation with the previously described parameters, animals infected by Beijing-genotype strains generally developed more pulmonary necrosis. Moreover, animals infected by strain 46 and vaccine-exposed strain 46P had a significantly different lung damage pattern (Figure 3). First, animals infected by vaccine-exposed strain 46P developed pneumonia earlier throughout the course of the disease, with initial small pneumonic areas appearing as early as day 14 postinfection compared with strain 46 (9.8 vs. 0%; *p* < 0.001), and gradually increasing throughout day 21 (20.9 vs. 13.2%; *p* < 0.01) and 28 (65.5 vs. 60.5%; *p* > 0.05) postinfection (Figure 3A). Although by day 28 the area affected by pneumonia was similar between animals infected by both strains, in the case of animals infected by strain 46P, these pneumonic areas were mostly occupied by areas of pulmonary necrosis, with large areas occupied by cellular debris and fragmented cells, along with an intensely acidophilic material in the extracellular space. When this parameter was measured, animals infected by strain 46P had a significantly larger lung percentage affected by necrosis at day 21 (11.6 vs. 2.5%; *p* < 0.05) and 28 (46.6 vs. 4.9%; *p* < 0.001) post-infection (Figure 3B). We also quantified the number and size of granulomas in animals infected by strains 46 and 46P (the strains which had showed significant differences in other virulence parameters) and identified that animals infected by vaccine-exposed strain 46P developed granulomas later compared with those infected with original strain 46 (average number of granulomas at days 7 [2 vs. 0; *p* < 0.05]; 14 [18.3 vs. 4.3; *p* < 0.001]; 21 [6.3 vs. 6.3; *p* > 0.05]; and 28 [3.0 vs. 1.3; *p* > 0.05]) (Figure 3C). Additionally, the size of the granulomas was significantly smaller in animals infected by strain 46P compared with those infected with strain 46 (average size of granulomas at days 7 [9655.6 vs. 0%; *p* < 0.001]; 14 [12977.6 vs. 4324.0%; *p* < 0.001]; 21 [17522.4 vs. 7423.4%; *p* < 0.001]; and 28 [14915.4 vs. 7970.0%; *p* < 0.001]) (Figure 3D). Representative images of the areas of necrosis, pneumonia and of granulomas in animals infected by strains 46 and 46P can be found in Figures 4, 5.

**Figure 6:**
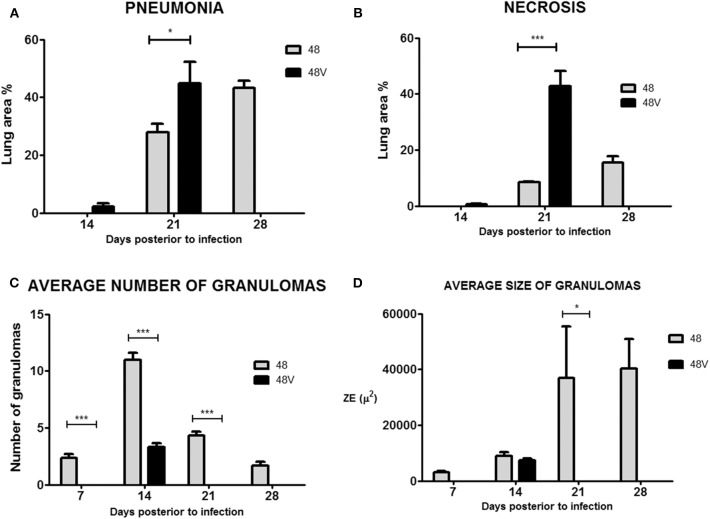
**(A)** Lung area affected by pneumonia in animals infected by strain 48 and 48V. **(B)** Lung area affected by necrosis in animals infected by strain 48 and 48V. **(C)** Average number of granulomas in animals infected by strain 48 and 48V. **(D)** Average size of granulomas in animals infected by strains 48 and 48V. *p < 0.05, ***p < 0.001.

**Figure 7:**
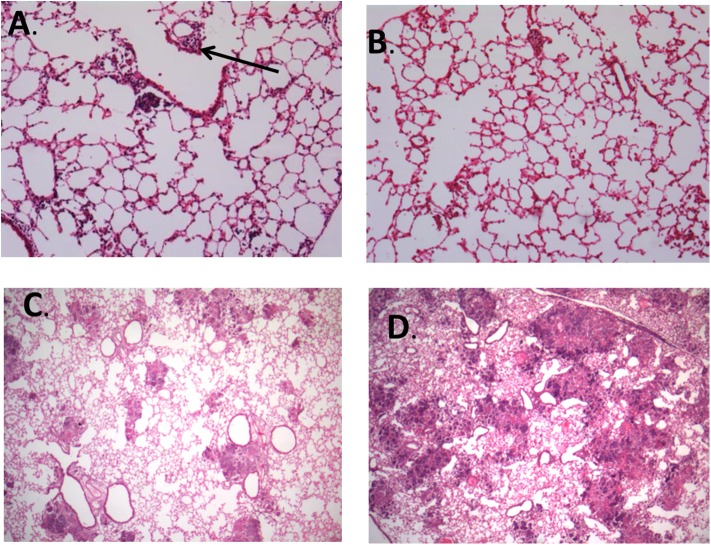
Representative images of histopathological findings in animals infected by strain 48 and strain 48V. **(A)** Lung tissue from animal infected by strain 48 at day 14 postinfection, with formation of small granulomas and perivascular inflammation (arrow) (100X magnification). **(B)** Lung tissue from animal infected by strain 48V at day 14 postinfection shows scarce interstitial inflammation without granuloma formation (100X magnification). **(C)** Animals infected by strain 48 had small pneumonic and necrotic areas throughout the lung tissue by day 21 postinfection (25X magnification). **(D)** Animals infected by strain 48V developed large confluent areas of pulmonary necrosis by day 21 postinfection (25X magnification). Stained with hematoxylin and eosin.

The histological parameters in animals infected by Beijing-genotype strain 48 and vaccine-exposed strains 48P, 48V, and 48S were similar to those identified in the experiments with strain 46. Animals infected by vaccine-exposed strain 48V developed pneumonia earlier and with more extensive lung area compromised compared with animals infected by strain 48 [days 14 [2.2 vs. 0%]; 21 [44.9 vs. 27.8%; *p* < 0.05]; 28 [animals infected by strain 48V all suffered spontaneous death by this day vs. 43.2%] postinfection, respectively] (Figure 6A). In terms of necrosis, animals infected by strain 48V developed small areas of necrosis by day 14 postinfection (0.5 vs. 0%; *p* > 0.05), which were significantly greater by day 21 postinfection compared with those found in animals infected by strain 48 (42.9 vs. 8.4%; *p* < 0.001) (Figure 6B). This comparison cannot be made for day 28 postinfection since all animals infected by strain 48V had been deceased by this day. Similar to strain 46P, animals infected by vaccine-exposed strain 48V had a statistically significantly smaller number and average size of granulomas throughout the course of the infection (Figures 6C,D). Representative images of the areas of necrosis, pneumonia and of granulomas in animals infected by strains 48 and 48V can be found in Figure 7 (animals infected with strain 48V all suffered spontaneous death before day 28 postinfection, therefore no images are available for the animals at this time point during the kinetics of the experiment).

### Transcriptomic Profile

**Figure 8:**
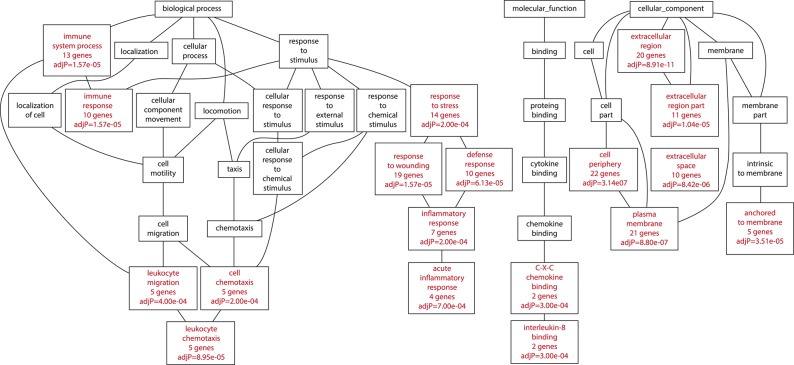
Biological processes impacted by up-regulated genes in animals infected with strain 46 compared with animals infected with strain 46P at day 21 postinfection.

**Figure 9:**
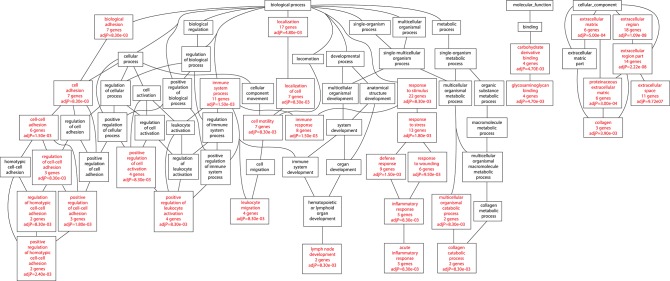
Biological processes impacted by up-regulated genes in animals infected with strain 48 compared with animals infected with strain 48V at day 21 postinfection.

Among those bacteria which showed a significantly different disease phenotype from the virulence experiments (46 vs. 46P; 48 vs. 48V) we isolated RNA from the lungs of animals at day 21 postinfection (this day in both cases showed the highest difference in terms of survival, lung bacillary load and histological damage between the aforementioned strains). We identified a total of 41 up-regulated genes in the mice infected by strain 46 compared with those infected by strain 46P at day 21 post-infection (Supplementary Table 2). Furthermore, a total of 45 up-regulated genes were identified in mice infected by strain 48 compared with those infected by strain 48V at day 21 post-infection (Supplementary Table 3). A total of 8 genes were down-regulated in animals infected by strain 46 compared with those infected by strain 46P (Supplementary Table 4) and 69 genes were downregulated in animals infected by strain 48 compared with those infected by strain 48V (Supplementary Table 5). Several biological processes were impacted with the up-regulation of genes relative to strains. For animals infected by strains 46 vs. 46P, up-regulated genes were significantly grouped in terms of immune-related processes, including genes related to immune response, leucocyte migration, leucocyte chemotaxis, response to stress, inflammatory response, acute inflammatory response, C-X-X chemokine binding, and interleukin-8 binding, among others (Figure 8). In the case of animals infected by strains 48 vs. 48V, up-regulated genes included those associated with immune processes, including immune system process, and immune response, leucocyte migration, positive regulation of leucocyte activation, inflammatory response, and acute inflammatory response. Additionally other pathways were also enriched in the animals infected by these strains, including the up-regulation of collagen catabolic processes and lymph node development (Figure 9).

We identified several influenced pathways with differential gene expression in mice infected by the different Beijing-genotype bacilli. For animals infected with strain 46 vs. 46P, four pathways were enriched in terms of up-regulated genes (complement and coagulation cascades [*n* = 4]; cytokine-cytokine receptor interaction [*n* = 5]; hematopoietic cell lineage [*n* = 3]; and toll-like receptor signaling pathway [*n* = 3]). For animals infected by strains 48 vs. 48V, three pathways were enriched in terms of up-regulated genes (amoebiasis [*n* = 3]; cytokine-cytokine receptor interaction [*n* = 4]; and osteoclast differentiation [*n* = 3]). Interestingly, when comparing the animals infected by both bacterial sets (46 vs. 46P, 48 vs. 48V) we identified two up-regulated genes which were present in both cases. These included Glycoprotein-2 and Vanin-1.

### Genomic Profile

When comparing the genome sequence from bacilli before and after passage through vaccinated mice, several mutations and other type of variants were observed. Variants were compared between the original strains and the vaccine-exposed strains (46 vs. 46P, 48 vs. 48V). Strain 46 had two unique mutations compared to strain 46P, while strain 46P had three mutations compared to strain 46. Strain 48 had five mutations compared to strain 48V, while strain 48V presented nine mutations compared to strain 48 (Table 2). Interestingly, when comparing the mutations present in both vaccine-exposed strains (46P vs. 48V) we identified one variant which was shared by both genomes. This variant was an inframe insertion present in a PE-family protein. The datasets for the sequenced genomes and lung transcriptomes in this project have been deposited as a Bioproject at NCBI and can be accessed through the project number PRJNA628024.

**Table 2:** Genome variants present in Beijing genotype bacilli before and after passage through BCG-vaccinated mice.

**Strain**	**Number of variants**	**Type of variant**	**Sequence change**	**Product**
46	2	Frameshift variant	c.545dupG p.Val183fs	Glycerol kinase
		Synonymous variant	c.169C>T p.Leu57Leu	ESAT-6-like protein EsxI
46P	3	Disruptive inframe insertion	c.3913_3914insGCACCGGCG p.Gly1304_Val1305insGlyThrGly	PE family protein
		Frameshift variant	c.1305_1306dupCC p.Gln436fs	PPE family protein PPE13
		Missense variant	c.812T>C p.Val271Ala	PE family protein
48	5	Conservative inframe insertion	c.2109_2110insGACGGTGGGGCCGGCGGCAACGGCGCC p.Ala703_Asn704insAspGlyGlyAlaGlyGlyAsnGlyAla	PE family protein
		Missense variant	c.3263G>T p.Gly1088Val	DNA-directed RNA polymerase subunit beta'
		Synonymous variant	c.1407_1410delTGGAinsCGGT p.471	PE family protein
		Synonymous variant	c.195C>T p.Ser65Ser	DUF222 domain-containing protein
		Synonymous variant	c.687T>C p.Gly229Gly	PE family protein
48V	9	Conservative inframe insertion	c.58_66dupTTGGCACTG p.Leu22_Ile23insLeuAlaLeu	LppP/LprE family lipoprotein
		Disruptive inframe deletion	c.483_500delCAACGGCGGCGCCGGCGG p.Asn162_Gly167del	hypothetical protein
		Disruptive inframe insertion	c.3913_3914insGCACCGGCG p.Gly1304_Val1305insGlyThrGly	PE family protein
		Frameshift variant and missense variant	c.4774_4777delAAGGinsGAGGT p.Lys1592fs	PE-PGRS family protein
		Missense variant	c.1416_1426delTGTGAGTAGTTinsCATCAACAGTA p.ValSerSerSer473IleAsnSerThr	Hypothetical protein
		Missense variant	c.3259_3263delCGGGGinsTGGGT p.ArgGly1087TrpVal	DNA-directed RNA polymerase subunit beta'
		Synonymous variant	c.1407T>C p.Gly469Gly	PE family protein
		Synonymous variant	c.1713T>C p.Gly571Gly	PE family protein
		Synonymous variant	c.1809A>T p.Ala603Ala	Hypothetical protein

## Discussion

The WHO estimates that in the last two decades 43 million lives have been saved by improved TB control. Despite the encouraging data, we are still far from reaching the ambitious goals of the END-TB strategy, which include reducing global mortality attributable to TB by 95% in the year 2030. The current global trend shows only a 2% reduction in the number of TB deaths per year; however, a 17% yearly reduction would be required to achieve the WHO target. As such, it is strikingly clear that ending TB will require “a surge in our efforts” (33). It has been almost a century since the BCG was introduced as, so far, the only vaccine to control TB (6), and although this was successful in several geographic regions, its limitations in terms of efficacy against pulmonary TB have become clear in the past decades. BCG vaccination has been shown to be highly cost-effective, and it has been consistently shown to prevent meningeal and miliary TB (34). Nonetheless, its ability to prevent pulmonary TB is much less well-established, particularly in the case of the Beijing genotype. It has been previously suggested that BCG could act as a selective pressure, favoring genetic and phenotypic changes which would in turn contribute to the dissemination of these conserved strains, however, experimental data to support this hypothesis had never before been presented (5).

In this study we present evidence stemming from a murine TB model, in which we directly evaluated the effect of BCG vaccination on the virulence of two Beijing genotype strains and a well-characterized MTB control strain, H37Rv. Interestingly, only Beijing-genotype strains (46 and 48) showed a significant increase in virulence after exposure to BCG-induced immunity. The presented data supports previous observations, in which Beijing-genotype strains have been identified as having adaptive advantages compared with other genotypes, including increased expression of α-crystallin and the *dosR* regulon gene, and induction of a deteriorated immune response in infected hosts (12, 35). Altogether, these characteristics lead to an increased virulence, evasion of host immune response and an increased capacity for latency and transmission (12).

As a consequence of vaccination, the immune system is primed to generate a significant arrangement of cells, cytokines and humoral factors to create a hostile environment against microorganisms. In the case of MTB, this pathogen can readily adapt to hostile environments including nutrient deficiency, high media acidity and even hypoxia (36). Despite the environment induced by BCG vaccination the infection, or even the disease, often cannot be prevented (22). During this process, the fittest bacteria may undergo adaptive evolution (37). Accordingly, mass vaccination may shift the previously existing balance between wild-type and vaccine-resistant strains to the latter (38). This is mainly the case when the level of induced immunity is inferior in terms of cross-reactivity compared with naturally-acquired immunity and the pathogen strain variability is high (38), such as with MTB (Supplementary Figure 2). Such shifts in bacterial populations, in order to “outsmart” their host have been previously studied (39). In a study performed in Dutch patients, researchers observed important polymorphisms in virulence factors of *Bordetella pertussis* after the introduction of whole-cell vaccination. Moreover, these polymorphisms allowed the bacteria to cause disease in hosts who had been previously vaccinated, since the polymorphisms conferred these vaccine-adapted strains with different virulence factors compared with those which were present in the strains used to prepare the vaccine (39). Interestingly, the authors in this study observed a statistically-significant inverse relation between the percentage of specific subtypes of the virulence factor P.69 present in the strains isolated from individuals, and their degree of vaccine-induced immunity. This finding highlights the fact that the shift in bacterial population from those which carry the identical virulence factor as in the vaccine strain (i.e., P.69A) to strains with non-vaccine-type P.69, has been very likely driven by vaccination. Thus, as previously discussed by van Loo Inge “like the use of antibiotics, vaccination can impose a strong selective force on populations of microorganisms, nevertheless long term, population-based, studies addressing the effect of vaccination are limited” (40). Among some of the earlier evidence, studies have reported that following the introduction of H. influenza vaccination, a small increase in incidence of invasive disease was observed (41). The population structure of the measles virus has likely also been affected by vaccination, and evidence shows that genes from pre-vaccination isolates have important variants which result in antigenic differences with pre-vaccination isolates as well as with vaccine-strain virus (42, 43). Recently, several mumps outbreaks have been identified in locations with high vaccine coverage (>95%), and results from molecular epidemiology studies suggest a higher prevalence of specific genotype viruses which have a substantial capacity to spread among vaccinated hosts. An epidemic wave reported in 2015 in Catalonia, for example, has been related to antigenic differences between the circulating and vaccine strains (termed immune escape) in addition to a waning immunity (44). Overall, results from different studies in a varied number of microorganisms suggest that circulating genotypes might be affected over time, and this in turn might advocate for a continuous evaluation of vaccine strain efficacy while considering the current molecular epidemiology scene.

Among the MTB genotype families the Beijing genotype is recognized for being particularly capable to evade the immune response (20). Some of these strains have managed, as reflected by multiple determinants, to cause serious illness in naïve as well as vaccinated individuals, creating a new risk in regions where TB had long ceased to be a serious public health threat (45). In this regard, one of the most interesting findings in our study is the fact that two MTB strains of the Beijing genotype showed a significant increase in virulence when exposed to vaccinated mice. This was not the case for the Euro-American genotype strain, highlighting the different response to vaccination amongst the different MTB lineages. Arguably, H37Rv is a lab adapted strain which might be unable to readily adapt under these circumstances. However, it is important to highlight that when both strains, 46 and 48, were exposed to sham-vaccinated mice, neither one of the sub strains (46S and 48S) had a hyper-virulent phenotype compared to the original-stock strain. This would suggest that the change in virulence is due to the vaccinated host, rather than other factors. Interestingly, the Beijing-genotype strains did not have the same response in terms of which BCG-substrain exposure produced differences in disease phenotype. In the case of strain 46, exposure to BCG-Phipps produced the higher-virulence strain: 46P; meanwhile in the case of strain 48, exposure to BCG-Vietnam produced the higher-virulence strain: 48V. Both BCG substrains were selected for this experiment because of previous characterization in which using laboratory strain H37Rv and other genotype strains, BCG-Phipps showed a higher degree of protective efficacy compared with BCG-Vietnam (46). Nonetheless, when these vaccinated animals were infected with strains 46 and 48, histopathological damage was different according to the infective strain and the BCG substrain used for immunization (Supplementary Figure 3). Interestingly, strain 46 was collected from the region of Vietnam, and therefore it is likely that this strain had historically been adapted to BCG-Vietnam vaccinated hosts, unlike strain 48, which was isolated from South Africa. The images would suggest that protection by BCG-Phipps is better in the case of strain 48, therefore likely leaving a higher number of escape variants in animals infected with BCG Vietnam, nonetheless this is a hypothesis and further studies are in course in order to characterize the factors underlying this observation. In such case, it is interesting to point out that, as previous studies have ascertained, BCG protection varies greatly according to vaccine substrain and also according to the particular infective strain, even among same-genotype isolates (46).

It is important to note that the initial experiments which evaluated virulence suggested a common mechanism by which the Beijing-genotype bacteria increased their virulence, as both strains 46P and 48V showed a similar virulence phenotype: both vaccine exposed strains showed similar survival rates, with 100% mortality 4 weeks post-infection; both vaccine-exposed strains produced extensive pulmonary necrosis instead of (or in addition to) the more common histopathological finding in this animal model (pneumonia), and both strains produced very little, if any, tissue damage and inflammation (perivascular and interstitial inflammation) and formation of granulomas during days 1-14 post-infection, as if going unrecognized by the host immune response (Additional images for histology patterns can be found in Supplementary Figures 4, 5). Nonetheless, the transcriptome pattern in lungs of animals infected by the Beijing-genotype strains differed considerably at day 21 post-infection. Animals infected by strains 46 and 46P differentially expressed several genes with known immune functions; for example the CD-14 antigen, a known co-receptor that binds microbial molecules in monocytes and macrophages. Previous studies using CD14 knockout mice infected with MTB have shown that these animals have a reduced inflammatory response, which protects them from lethality through the late course of infection (47). In the case of animals infected by strains 48 and 48V, differentially expressed genes included cytokine-like 1, a known chemotactic factor for monocytes and macrophages. This cytokine is similar to CCL2, which is known to increase macrophage and T cell accumulation in the lung, as well as mediate their organization during MTB infection (48), a finding which would suggest an explanation to the difference in early-phase inflammation in the lungs of these animals. Other differentially expressed genes in the lungs of animals infected by strains 46 and 46P included IL-33 as well as Interleukin 1 receptor (type 1). In the specific case of MTB, IL-33 has been shown to have protective effects in infected animals, as well as a therapeutic effect when given systemically post-infection (49). Interestingly, two genes were up-regulated in both Beijing-genotype infected animals, glycoprotein 2, which plays an important role in the innate immune response, and vanin 1, which in mice regulates the migration of T-cell progenitors to the thymus. Although the role of these factors during MTB infection remains unexplored, it is interesting to note that Vanin-1 has been previously studied in models with other microorganisms. In a previous study, vanin-1 was shown to control granuloma formation and macrophage polarization during *Coxiella burnetii* infection (50). In this study, the authors conclude that vanin-1 is a key component in shaping the nature and intensity of the immune response against infection, this derived from the fact that vanin-1 has a role in macrophage polarization toward an M2 phenotype, leading to a defective granuloma formation and reduced microbicidal response. Although this role in MTB infected mice has not been specifically studied, our results show that animals infected by these strains have a differential expression of Vanin-1 (both in the case of vaccine-exposed strains 46P and 48V), and this correlates with a decreased ability by the animals infected with these strains to form granulomas during the early phase of the disease (day 14) compared with animals infected by the original stock-strains (Supplementary Figure 6), and which limits granuloma size during the later stage (days 21–28). These results warrant future investigation into the role of Vanin-1 during MTB infection, and its potential therapeutic effect as an immune-modulator. It is important to highlight that the fold-change in these differentially-expressed genes ranged to 1.44 in the upper limit. Nonetheless, such changes in previous studies have been reported to provide clinically meaningful information (51), and therefore the biological significance of such changes cannot at this point be conclusively ascertained.

Lastly, with DNA sequencing we found virulence associated genetic mutations in vaccine-exposed strains compared with the original-stock strains. At this point, this data seems to support the hypothesis that these mutations are the result of selective pressure by the host's immune system, however, it is important to note that they might also be caused by random mechanisms, so further exploration should be considered before drawing conclusions. Although we found one shared mutation (Disruptive inframe insertion c.3913_3914insGCACCGGCG p.Gly1304_Val1305insGlyThrGly) between both hyper-virulent vaccine-exposed strains, it is at this point difficult to evaluate the potential biological effect of this in-frame insertion. Furthermore, this variant was observed in a sequence which encodes a PE-protein. PE along with PPE proteins make up to 10% of the coding regions in MBT genomes, and as can be seen in Table 2, several mutations identified fall in these coding regions. These proteins share a proline-glutamic acid (PE) or proline-proline-glutamic acid (PPE) motif, and although their specific function remains unknown, several authors suggest that they provide antigenic variation to the bacteria. The sequences are of a highly polymorphic nature, and therefore the biological relevance of the mutation present in both these strains should be further explored.

Overall, the experiments presented in this study present experimental evidence as to the role of vaccinated hosts in shaping the host-pathogen interactions. From our results, it could be suggested that hosts interact differently with pathogens with different degrees of virulence, despite their having a very similar genomic make-up. Pathogens on the other hand, are constantly adapting to a changing environment, and can alter how they are recognized by the host's immune system in order to survive in a vaccinated population. Nonetheless, we urge a cautious interpretation of these results, and call attention to the fact that this data stems from a murine TB model, and should therefore not be extrapolated to human populations at this stage. Nonetheless, this information could be considered in the urgent need to produce a higher-efficacy vaccine against TB. As in the case of *Bordetella pertussis*, it could be possible that we can counter the spread of these vaccine-adapted strains by introducing a different prophylactic strategy to which they have not yet developed adaptive mechanisms (39, 52, 53). The study presented here has several strengths, including the use of syngeneic mice in order to reduce inter-host variance, and the use of methodology to avoid artifacts during the genome comparison process, including the use of PCR-free methods to build the DNA-libraries for sequencing, as well as a depth of sequencing ranging from 475 to 715 in each of the sequenced strains. Nonetheless, it is important to highlight that the results should be interpreted in light of the limitations of the study, including a design which did not consider the sequencing of strains which did not portray a virulence change in the initial stage of the study (i.e., strains 46V, 48P, 46S, and 48S), and the fact that in this particular study only two MTB Beijing genomes were tested. Currently, further studies are underway which seek to evaluate this effect on the wide MTB genomic landscape, which should give more information and add valuable information. Last, it is important to once again highlight that the results from this murine model should provide information, but we do not encourage extrapolation of these results to the clinical setting. Rather, we are currently working to ascertain the effects of BCG vaccination on bacteria recovered from vaccinated and non-vaccinated individuals from a single transmission chain. Further information is needed using other experimental models in order to draw more robust conclusions.

As a closing remark, we emphatically stress that vaccination is currently the most cost-effective health intervention, and saves an estimated three million lives per year. However, BCG vaccination as the only prophylactic strategy approved by the WHO falls short of the current epidemiological needs, and therefore considerable efforts, both financial and intellectual, should be put forward in order to seek a more effective strategy, meanwhile optimal use of BCG as recommended by the WHO remains adamantly needed in light of the current world trends regarding tuberculosis incidence and mortality.

## Data Availability Statement

The datasets for the sequenced genomes and lung transcriptomes in this project have been deposited as a Bioproject at NCBI and can be accessed through the project number PRJNA628024. All other datasets will be made available by request to the corresponding author without any restriction.

## Ethics Statement

The animal study was reviewed and approved by Animal Experimentation Committee at the National Institute of Medical Sciences and Nutrition México (PAT-1860-16/18-1).

## Author's Note

Partial results from this study were presented at the Keystone Symposia held in Santa Fe, New Mexico, January 22–January 27, 2015. Abstract number: 3079.

## Author Contributions

ZZ-B performed the in vivo experiments, including mice euthanasia, CFU determination, lung tissue preparation and assessment of lung pathology. Additionally, she performed the molecular biology experiments (including RNA-seq and DNA-seq), and participated in the data analysis and interpretation, as well as manuscript writing and revision. OR-E performed molecular biology experiments and lung tissue preparation and assessment of lung pathology. DM-E planned, coordinated the study, prepared the bacterial strains and vaccines, determined lung bacillary load and analyzed and interpreted the data. BM-C aided in the performance of the molecular biology experiments. JB-P infected the mice and kept survival records. OM-L performed the bioinformatics analysis for the genomic data. FC-G and AO-L performed the genomic experiments and contributed with the data analysis. CM-R performed in vivo experiments including mice euthanasia and CFU determination. ID and RR-V performed the RNA and DNA libraries and the NGS. GL-L analyzed the RNA-Seq data and performed the GO and KEGG-pathways enrichments. RW donated the MTB bacilli for the experiments, and critically reviewed the manuscript. RH-P, RA, and DS planned and coordinated the study, interpreted the data, and critically reviewed the manuscript. All authors reviewed the manuscript prior to submission and approved the final version.

## Conflict of Interest

The authors declare that the research was conducted in the absence of any commercial or financial relationships that could be construed as a potential conflict of interest.

## Supplementary Material

Supplementary Figure 1 **(A)** Survival rate of animals infected with the original stock strain 1 (H37Rv) and vaccine-exposed strains 1P, 1V, and 1S. **(B)** Lung bacillary load in animals infected with the original stock strain 1 and vaccine exposed strains 1P, 1V, and 1S. **(C)** Percentage of lung area affected by pneumonia in animals infected with the original stock strain 1 and vaccine-exposed strains 1P, 1V, and 1S. ^*^*p* < 0.05, ^**^*p* < 0.01.

Supplementary Figure 2 Emergence of vaccine-adapted microorganisms.

Supplementary Figure 3 **(A)** BCG Phipps vaccinated animals infected by strain 46 presented by 2 months post-infection moderate areas of pulmonary pneumonia **(B)** BCG Vietnam vaccinated animals infected by strain 46 presented by 2 months post-infection moderate areas of pulmonary pneumonic areas. **(C)** BCG Phipps vaccinated animals infected by strain 48 had small pneumonic areas by 2 months postinfection, suggesting a good level of protection by this BCG substrain compared with **(D)** BCG Vietnam vaccinated animals infected with strain 48 show large areas of pneumonia by 2 months postinfection, highlighting a lower level of protection against this Beijing-genotype strain (48). Stained with haematoxylin and eosin, images taken at 25X.

Supplementary Figure 4 Representative histological images from lungs of animals infected with strains 46, 46P, 46V, and 46S and euthanized on days 14, 21, and 28 post-infection. Animals infected by strain 46P show large areas of necrosis (^*^) compared with animals infected with the original-stock strain 46, or animals infected with strains 46V and 46S, which develop lung damage in the form of pneumonia (arrows). Stained with haematoxylin and eosin (Black bar in the first image corresponds to 500 μm).

Supplementary Figure 5 Representative histological images from lungs of animals infected with strains 48, 48P, 48V, and 48S and euthanized on days 14, 21, and 28 post-infection. Lungs from animals infected with the original-stock strain 48 show interstitial, peribronchial, and perivascular inflammation (^*^) by day 14 post-infection. Contrary, lungs from animals infected by strain 48V show very scarce if any inflammatory infiltrate on day 14 post-infection. Lungs from animals which were infected by strain 48V and euthanized on day 21 post-infection show pneumonic and necrotic areas (arrows), in comparison with those infected by strain 48 on this same time-point, which show mostly pneumonia (dotted arrow), with very scarce or null necrosis. Stained with hematoxyylin and eosin (black bar in the first image corresponds to 500 μm).

Supplementary Figure 6 Representative granuloma from a subject infected by original stock strain 46, and sacrificed at day 7 postinfection. Stained with hematoxylin and eosin, 100X magnification.

Supplementary Table 1 Genome assembly data.

Supplementary Table 2 Up-regulated genes in the mice infected by strain 46 compared to those infected by strain 46P.

Supplementary Table 3 Up-regulated genes in the mice infected by strain 48 compared to those infected by strain 48V.

Supplementary Table 4 Down-regulated genes in the mice infected by strain 46 compared to those infected by strain 46P.

Supplementary Table 5 Down-regulated genes in the mice infected by strain 48 compared to those infected by strain 48V.
