# Supplementary figures and images for: Evidence for the Effect of Vaccination on Host-Pathogen Interactions in a Murine Model of Pulmonary Tuberculosis by Mycobacterium tuberculosis
Source: Front Immunol. 2020 May 19;11:930. doi: 10.3389/fimmu.2020.00930 (PMC7248268; doi:10.3389/fimmu.2020.00930)

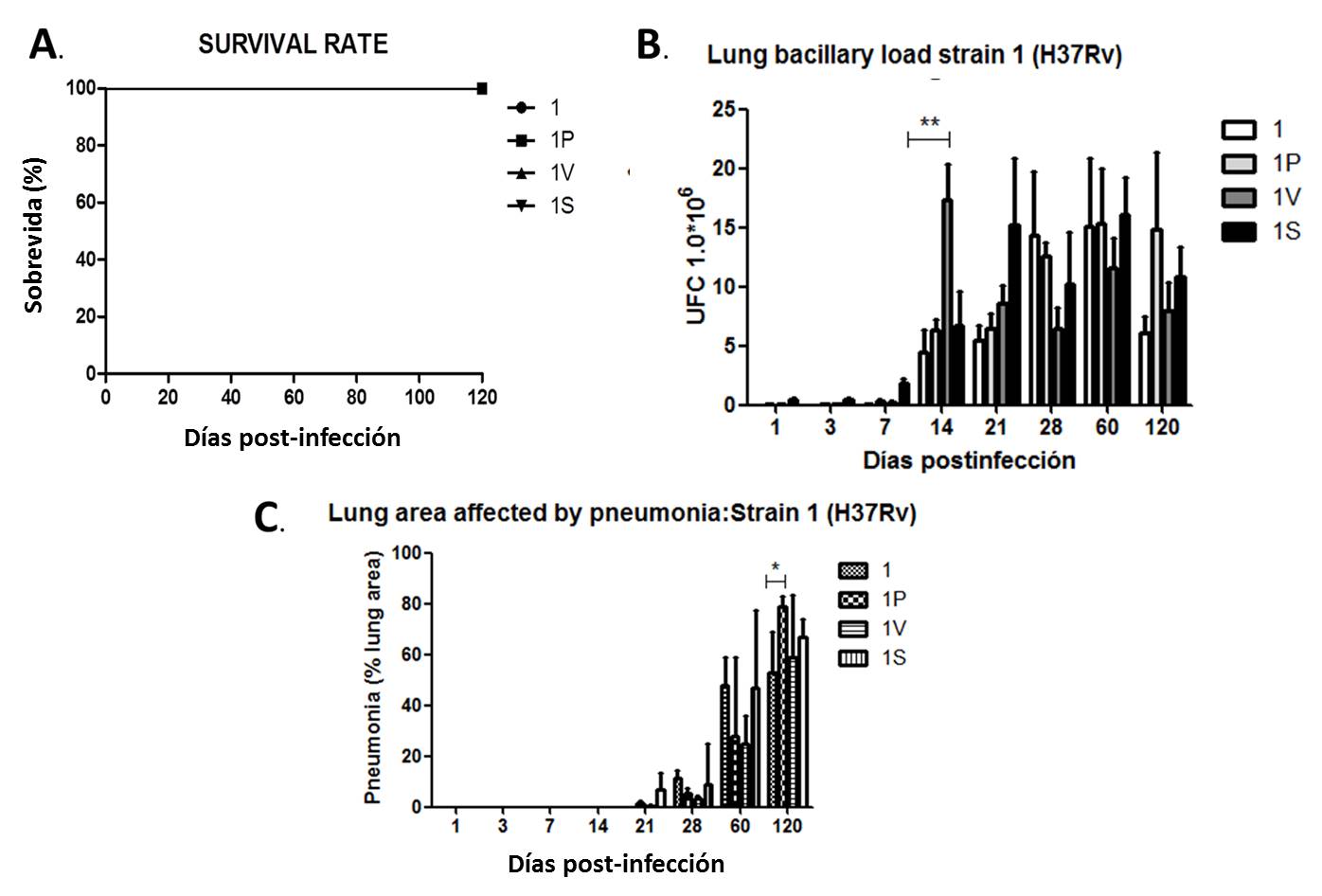

Supplement: Supplementary Figure 1 — (A) Survival rate of animals infected with the original stock strain 1 (H37Rv) and vaccine-exposed strains 1P, 1V, and 1S. (B) Lung bacillary load in animals infected with the original stock strain 1 and vaccine exposed strains 1P, 1V, and 1S. (C) Percentage of lung area affected by pneumonia in animals infected with the original stock strain 1 and vaccine-exposed strains 1P, 1V, and 1S. *p < 0.05, **p < 0.01. [file Image_1.JPEG]

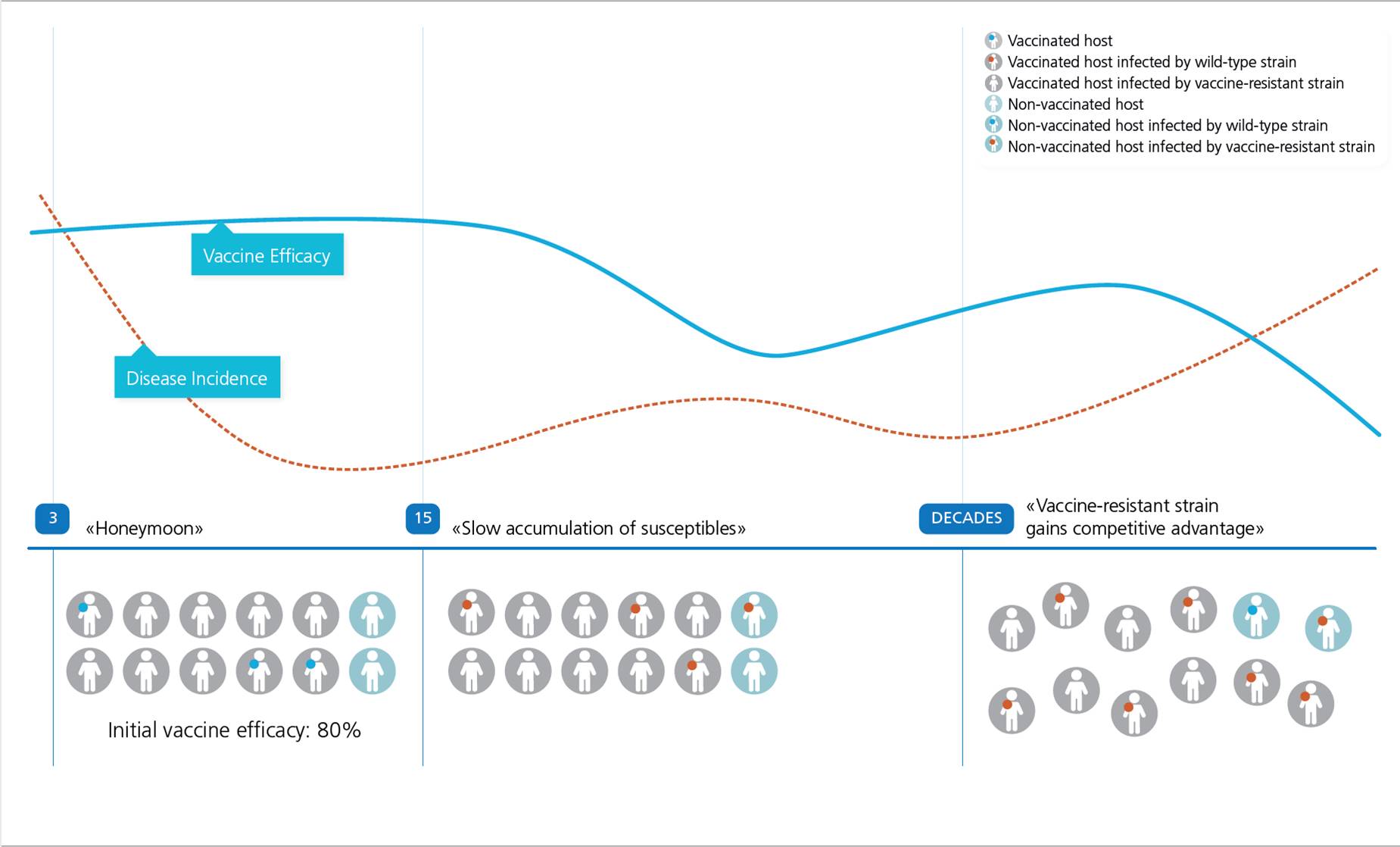

Supplement: Supplementary Figure 2 — Emergence of vaccine-adapted microorganisms. [file Image_2.JPEG]

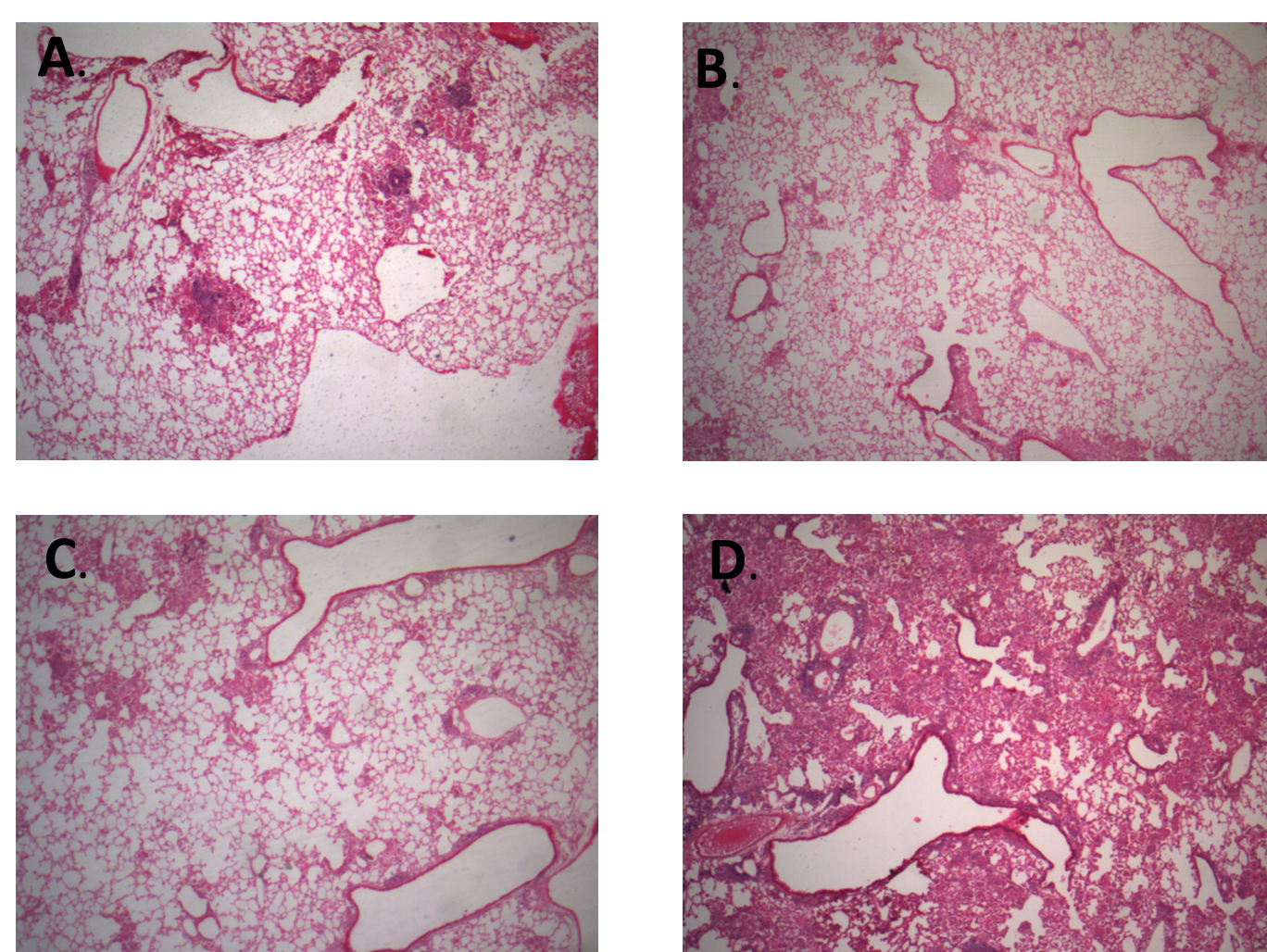

Supplement: Supplementary Figure 3 — (A) BCG Phipps vaccinated animals infected by strain 46 presented by 2 months post-infection moderate areas of pulmonary pneumonia (B) BCG Vietnam vaccinated animals infected by strain 46 presented by 2 months post-infection moderate areas of pulmonary pneumonic areas. (C) BCG Phipps vaccinated animals infected by strain 48 had small pneumonic areas by 2 months postinfection, suggesting a good level of protection by this BCG substrain compared with (D) BCG Vietnam vaccinated animals infected with strain 48 show large areas of pneumonia by 2 months postinfection, highlighting a lower level of protection against this Beijing-genotype strain (48). Stained with haematoxylin and eosin, images taken at 25X. [file Image_3.TIF]

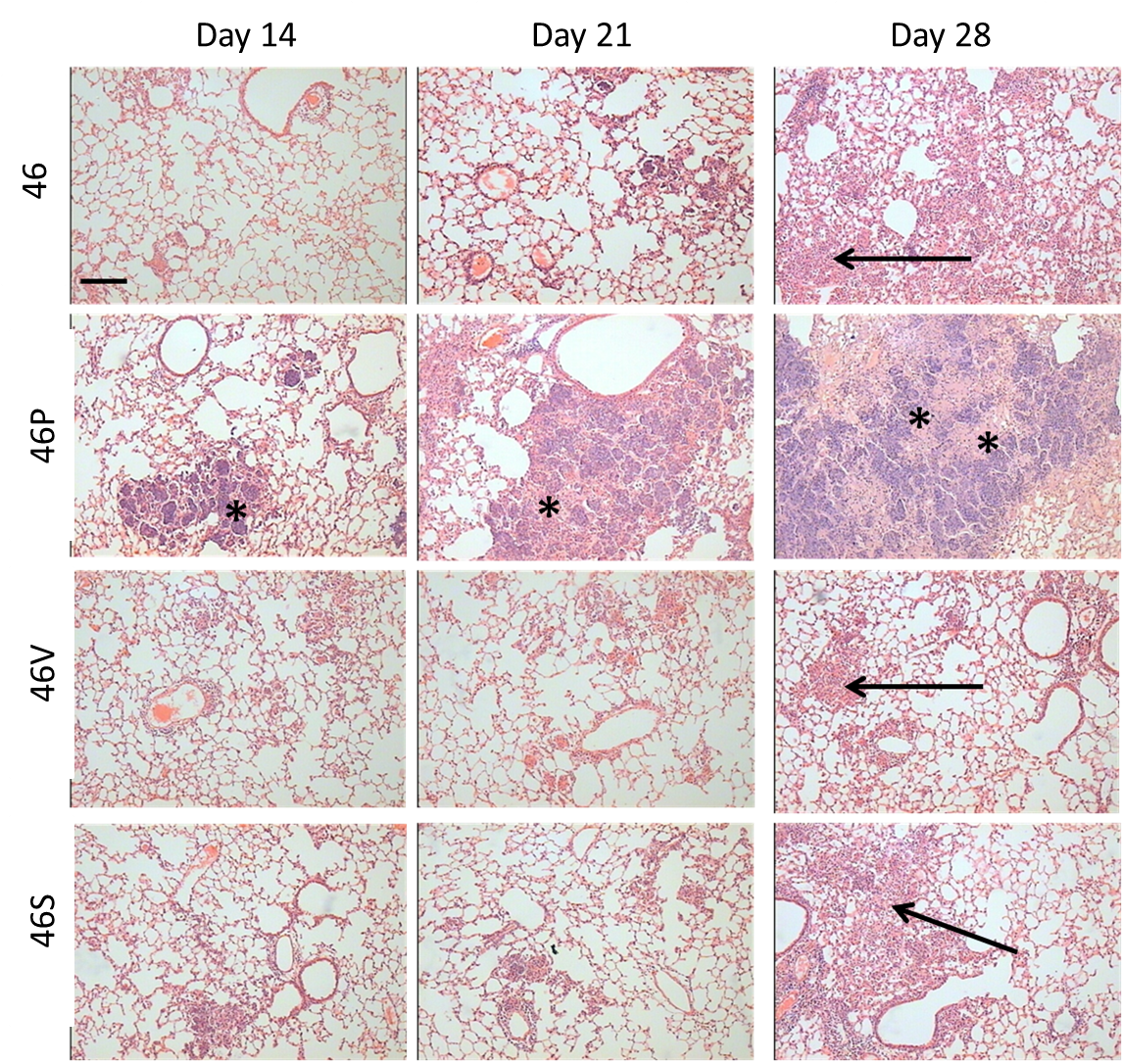

Supplement: Supplementary Figure 4 — Representative histological images from lungs of animals infected with strains 46, 46P, 46V, and 46S and euthanized on days 14, 21, and 28 post-infection. Animals infected by strain 46P show large areas of necrosis (*) compared with animals infected with the original-stock strain 46, or animals infected with strains 46V and 46S, which develop lung damage in the form of pneumonia (arrows). Stained with haematoxylin and eosin (Black bar in the first image corresponds to 500 μm). [file Image_4.TIF]

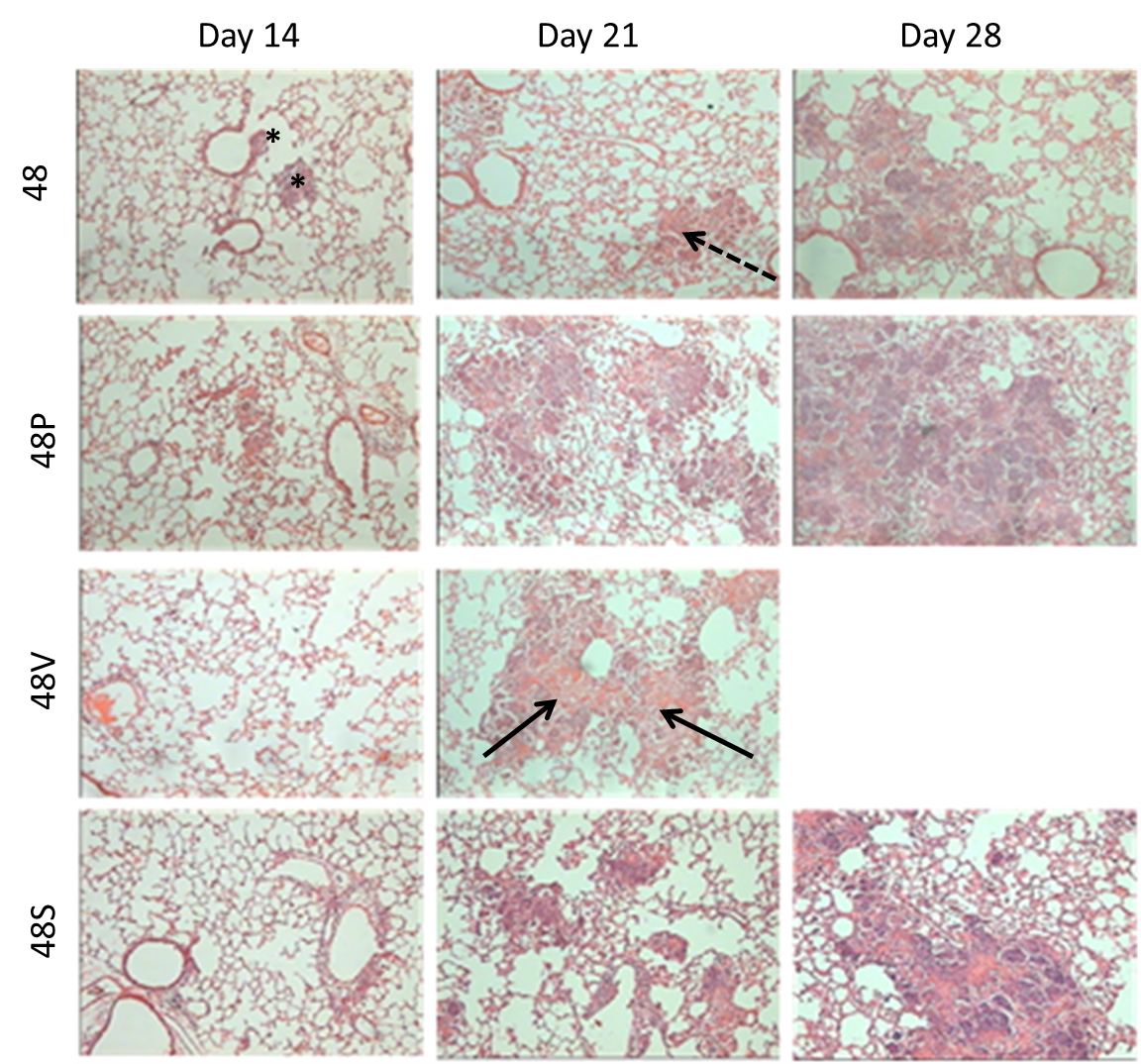

Supplement: Supplementary Figure 5 — Representative histological images from lungs of animals infected with strains 48, 48P, 48V, and 48S and euthanized on days 14, 21, and 28 post-infection. Lungs from animals infected with the original-stock strain 48 show interstitial, peribronchial, and perivascular inflammation (*) by day 14 post-infection. Contrary, lungs from animals infected by strain 48V show very scarce if any inflammatory infiltrate on day 14 post-infection. Lungs from animals which were infected by strain 48V and euthanized on day 21 post-infection show pneumonic and necrotic areas (arrows), in comparison with those infected by strain 48 on this same time-point, which show mostly pneumonia (dotted arrow), with very scarce or null necrosis. Stained with hematoxyylin and eosin (black bar in the first image corresponds to 500 μm). [file Image_5.TIF]

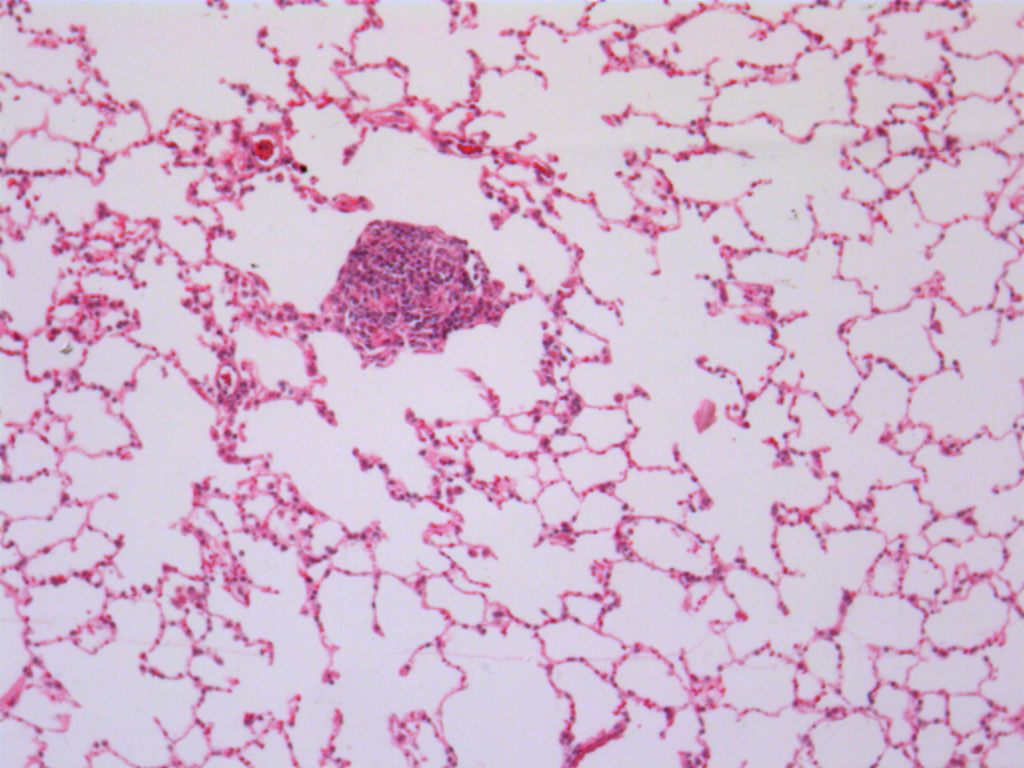

Supplement: Supplementary Figure 6 — Representative granuloma from a subject infected by original stock strain 46, and sacrificed at day 7 postinfection. Stained with hematoxylin and eosin, 100X magnification. [file Image_6.TIF]
