# Supplementary material for: Evidence for the Effect of Vaccination on Host-Pathogen Interactions in a Murine Model of Pulmonary Tuberculosis by Mycobacterium tuberculosis
Source: Front Immunol. 2020 May 19;11:930. doi: 10.3389/fimmu.2020.00930 (PMC7248268; doi:10.3389/fimmu.2020.00930)
Supplement: Supplementary Table 1 — Genome assembly data. [file Table_1.DOCX]

**Supplementary Table 1.** Genome assembly data

| **Assembly** | **Number of contigs** | **Coverage (Depth)** | **Assembly size (bp)** | **Largest contig (bp)** | **N50 (bp)** | **N90 (bp)** |
| --- | --- | --- | --- | --- | --- | --- |
| **46** | 71 | 475.56 | 4362907 | 302594 | 151200 | 54709 |
| **46P** | 83 | 715.25 | 4359576 | 303349 | 135482 | 47066 |
| **48** | 86 | 663.31 | 4359701 | 303331 | 151128 | 42231 |
| **48V** | 81 | 479.49 | 4359576 | 294930 | 137687 | 49723 |
