# Supplementary material for: Evidence for the Effect of Vaccination on Host-Pathogen Interactions in a Murine Model of Pulmonary Tuberculosis by Mycobacterium tuberculosis
Source: Front Immunol. 2020 May 19;11:930. doi: 10.3389/fimmu.2020.00930 (PMC7248268; doi:10.3389/fimmu.2020.00930)
Supplement: Supplementary Table 2 — Up-regulated genes in the mice infected by strain 46 compared to those infected by strain 46P. [file Table_2.DOCX]

**Supplementary Table 2.** Up-regulated genes in the mice infected by strain 46 compared to those infected by strain 46P

| **Gene-Id** | **LogFold**  **Change** | **padjust** | **Gi** | **Locus** | **Description** |
| --- | --- | --- | --- | --- | --- |
| gene15521 | 1.339835633 | 0.002652801 | 11689 | NC_000072.6 | gene_id=gene15521;Dbxref=GeneID:11689,MGI:MGI:87999;Name=Alox5;description=arachidonate 5-lipoxygenase;gbkey=Gene;gene=Alox5;gene_biotype=protein_coding;gene_synonym=5-LO,5-LOX,5LO,5LX,AI850497,F730011J02 |
| gene28273 | 1.44939259 | 0.002652801 | 276952 | NC_000077.6 | gene_id=gene28273;Dbxref=GeneID:276952,MGI:MGI:2685575;Name=Rasl10b;description=RAS-like%2C family 10%2C member B;gbkey=Gene;gene=Rasl10b;gene_biotype=protein_coding;gene_synonym=B230331P10Rik,Gm729,VTS58635 |
| gene15617 | 1.277881819 | 0.012371792 | 232345 | NC_000072.6 | gene_id=gene15617;Dbxref=GeneID:232345,MGI:MGI:2449119;Name=A2m;description=alpha-2-macroglobulin;gbkey=Gene;gene=A2m;gene_biotype=protein_coding;gene_synonym=A2mp |
| gene32151 | 1.32365106 | 0.012371792 | 74145 | NC_000079.6 | gene_id=gene32151;Dbxref=GeneID:74145,MGI:MGI:1921395;Name=F13a1;description=coagulation factor XIII%2C A1 subunit;gbkey=Gene;gene=F13a1;gene_biotype=protein_coding;gene_synonym=1200014I03Rik,AI462306,F13a |
| gene41262 | 1.329349765 | 0.012371792 | 72432 | NC_000084.6 | gene_id=gene41262;Dbxref=GeneID:72432,MGI:MGI:1919682;Name=Spink5;description=serine peptidase inhibitor%2C Kazal type 5;gbkey=Gene;gene=Spink5;gene_biotype=protein_coding;gene_synonym=2310065D10Rik,AV238912,LEKT1,LETK1,VAKT1 |
| gene12427 | 1.270643756 | 0.012389835 | 20750 | NC_000071.6 | gene_id=gene12427;Dbxref=GeneID:20750,MGI:MGI:98389;Name=Spp1;description=secreted phosphoprotein 1;gbkey=Gene;gene=Spp1;gene_biotype=protein_coding;gene_synonym=2AR,Apl-1,BNSP,Bsp,BSPI,Eta,ETA-1,OP,Opn,Opnl,Ric,Spp-1 |
| gene16776 | 1.240596242 | 0.013392163 | 12273 | NC_000073.6 | gene_id=gene16776;Dbxref=GeneID:12273,MGI:MGI:88232;Name=C5ar1;description=complement component 5a receptor 1;gbkey=Gene;gene=C5ar1;gene_biotype=protein_coding;gene_synonym=C5aR,C5r1,Cd88,D7Msu1 |
| gene2215 | 1.240314451 | 0.013392163 | 240913 | NC_000067.6 | gene_id=gene2215;Dbxref=GeneID:240913,MGI:MGI:1339949;Name=Adamts4;description=a disintegrin-like and metallopeptidase (reprolysin type) with thrombospondin type 1 motif%2C 4;gbkey=Gene;gene=Adamts4;gene_biotype=protein_coding;gene_synonym=3830423K05,ADAM-TS4,ADAMTS-2,ADMP-1,mKIAA0688 |
| gene875 | 1.264559952 | 0.013392163 | 12765 | NC_000067.6 | gene_id=gene875;Dbxref=GeneID:12765,MGI:MGI:105303;Name=Cxcr2;description=chemokine (C-X-C motif) receptor 2;gbkey=Gene;gene=Cxcr2;gene_biotype=protein_coding;gene_synonym=CD128,CDw128,Cmkar2,Gpcr16,IL-8rb,IL-8Rh,IL8RA,Il8rb,mIL-8RH |
| gene7757 | 1.252762079 | 0.013889128 | 12655 | NC_000069.6 | gene_id=gene7757;Dbxref=GeneID:12655,MGI:MGI:1330860;Name=Chil3;description=chitinase-like 3;gbkey=Gene;gene=Chil3;gene_biotype=protein_coding;gene_synonym=AI505981,Chi3l3,ECF-L,Ym1 |
| gene39522 | 1.243194037 | 0.02207697 | 378460 | NC_000083.6 | gene_id=gene39522;Dbxref=GeneID:378460,MGI:MGI:3576625;Name=Pram1;description=PML-RAR alpha-regulated adaptor molecule 1;gbkey=Gene;gene=Pram1;gene_biotype=protein_coding;gene_synonym=AY665714 |
| gene425 | 1.221372567 | 0.02207697 | 16178 | NC_000067.6 | gene_id=gene425;Dbxref=GeneID:16178,MGI:MGI:96546;Name=Il1r2;description=interleukin 1 receptor%2C type II;gbkey=Gene;gene=Il1r2;gene_biotype=protein_coding;gene_synonym=CD121b,Il1r-2 |
| gene26476 | 1.126853258 | 0.024534182 | 58223 | NC_000076.6 | gene_id=gene26476;Dbxref=GeneID:58223,MGI:MGI:1927899;Name=Mmp19;description=matrix metallopeptidase 19;gbkey=Gene;gene=Mmp19;gene_biotype=protein_coding |
| gene26627 | 1.201007176 | 0.024534182 | 18413 | NC_000077.6 | gene_id=gene26627;Dbxref=GeneID:18413,MGI:MGI:104749;Name=Osm;description=oncostatin M;gbkey=Gene;gene=Osm;gene_biotype=protein_coding;gene_synonym=OncoM |
| gene35454 | 1.151659926 | 0.024534182 | 16365 | NC_000080.6 | gene_id=gene35454;Dbxref=GeneID:16365,MGI:MGI:103206;Name=Acod1;description=aconitate decarboxylase 1;gbkey=Gene;gene=Acod1;gene_biotype=protein_coding;gene_synonym=AI323667,CAD,Irg1 |
| gene19818 | 1.126876063 | 0.026716439 | 11501 | NC_000073.6 | gene_id=gene19818;Dbxref=GeneID:11501,MGI:MGI:107825;Name=Adam8;description=a disintegrin and metallopeptidase domain 8;gbkey=Gene;gene=Adam8;gene_biotype=protein_coding;gene_synonym=ADAM 8,CD156,CD156a,E430039A18Rik,MS2 |
| gene29395 | 1.080343954 | 0.026716439 | 80879 | NC_000077.6 | gene_id=gene29395;Dbxref=GeneID:80879,MGI:MGI:1933438;Name=Slc16a3;description=solute carrier family 16 (monocarboxylic acid transporters)%2C member 3;gbkey=Gene;gene=Slc16a3;gene_biotype=protein_coding;gene_synonym=Mct3,Mct4 |
| gene37268 | 1.145243046 | 0.026716439 | 18022 | NC_000081.6 | gene_id=gene37268;Dbxref=GeneID:18022,MGI:MGI:97308;Name=Nfe2;description=nuclear factor%2C erythroid derived 2;gbkey=Gene;gene=Nfe2;gene_biotype=protein_coding;gene_synonym=NF-E2,NF-E2/P45,p45,p45nf-e2,p45NFE2 |
| gene12956 | 1.139251463 | 0.033913097 | 80885 | NC_000071.6 | gene_id=gene12956;Dbxref=GeneID:80885,MGI:MGI:1933383;Name=Hcar2;description=hydroxycarboxylic acid receptor 2;gbkey=Gene;gene=Hcar2;gene_biotype=protein_coding;gene_synonym=Gpr109a,Gpr109b,HM74,mHM74b,Niacr1,PUMA-G,Pumag |
| gene16262 | 1.131192612 | 0.033913097 | 245126 | NC_000073.6 | gene_id=gene16262;Dbxref=GeneID:245126,MGI:MGI:2442280;Name=Tarm1;description=T cell-interacting%2C activating receptor on myeloid cells 1;gbkey=Gene;gene=Tarm1;gene_biotype=protein_coding;gene_synonym=9930022N03Rik,ENSMUSG00000053338,Gm9904,OLT-2 |
| gene28288 | 1.150664503 | 0.033913097 | 20303 | NC_000077.6 | gene_id=gene28288;Dbxref=GeneID:20303,MGI:MGI:98261;Name=Ccl4;description=chemokine (C-C motif) ligand 4;gbkey=Gene;gene=Ccl4;gene_biotype=protein_coding;gene_synonym=Act-2,AT744.1,MIP-1B,Mip1b,Scya4 |
| gene35339 | 1.180860486 | 0.033913097 | 380924 | NC_000080.6 | gene_id=gene35339;Dbxref=GeneID:380924,MGI:MGI:2685142;Name=Olfm4;description=olfactomedin 4;gbkey=Gene;gene=Olfm4;gene_biotype=protein_coding;gene_synonym=GC1,Gm296,Gm913,GW112,OlfD,pPD4 |
| gene8097 | 1.0291375 | 0.033913097 | 74442 | NC_000069.6 | gene_id=gene8097;Dbxref=GeneID:74442,MGI:MGI:1921692;Name=Sgms2;description=sphingomyelin synthase 2;gbkey=Gene;gene=Sgms2;gene_biotype=protein_coding;gene_synonym=4933405A16Rik,5133401H06Rik,AI854299 |
| gene40162 | 1.059776598 | 0.034118837 | 76905 | NC_000083.6 | gene_id=gene40162;Dbxref=GeneID:76905,MGI:MGI:1924155;Name=Lrg1;description=leucine-rich alpha-2-glycoprotein 1;gbkey=Gene;gene=Lrg1;gene_biotype=protein_coding;gene_synonym=1300008B03Rik,2310031E04Rik,Lrg,Lrhg |
| gene16282 | 1.16482019 | 0.034153564 | 100038909 | NC_000073.6 | gene_id=gene16282;Dbxref=GeneID:100038909,MGI:MGI:3709645;Name=Gm14548;description=predicted gene 14548;gbkey=Gene;gene=Gm14548;gene_biotype=protein_coding;gene_synonym=OTTMUSG00000017169 |
| gene24743 | 1.134862614 | 0.034153564 | 22361 | NC_000076.6 | gene_id=gene24743;Dbxref=GeneID:22361,MGI:MGI:108395;Name=Vnn1;description=vanin 1;gbkey=Gene;gene=Vnn1;gene_biotype=protein_coding;gene_synonym=V-1 |
| gene6670 | 0.872321714 | 0.034153564 | 26570 | NC_000069.6 | gene_id=gene6670;Dbxref=GeneID:26570,MGI:MGI:1347355;Name=Slc7a11;description=solute carrier family 7 (cationic amino acid transporter%2C y+ system)%2C member 11;gbkey=Gene;gene=Slc7a11;gene_biotype=protein_coding;gene_synonym=9930009M05Rik,AI451155,sut,xCT |
| gene17133 | 1.020773438 | 0.034427995 | 18793 | NC_000073.6 | gene_id=gene17133;Dbxref=GeneID:18793,MGI:MGI:97612;Name=Plaur;description=plasminogen activator%2C urokinase receptor;gbkey=Gene;gene=Plaur;gene_biotype=protein_coding;gene_synonym=Cd87,u-PAR,uPAR |
| gene43019 | 1.120281196 | 0.034427995 | 240672 | NC_000085.6 | gene_id=gene43019;Dbxref=GeneID:240672,MGI:MGI:2685183;Name=Dusp5;description=dual specificity phosphatase 5;gbkey=Gene;gene=Dusp5;gene_biotype=protein_coding;gene_synonym=Gm337 |
| gene14047 | 1.102887146 | 0.03720559 | 338523 | NC_000072.6 | gene_id=gene14047;Dbxref=GeneID:338523,MGI:MGI:2443388;Name=Kdm7a;description=lysine (K)-specific demethylase 7A;gbkey=Gene;gene=Kdm7a;gene_biotype=protein_coding;gene_synonym=A630082K20Rik,BB041802,ENSMUSG00000073143,Jhdm1d,mKIAA1718 |
| gene17151 | 1.144677418 | 0.03720559 | 26366 | NC_000073.6 | gene_id=gene17151;Dbxref=GeneID:26366,MGI:MGI:1347248;Name=Ceacam10;description=carcinoembryonic antigen-related cell adhesion molecule 10;gbkey=Gene;gene=Ceacam10;gene_biotype=protein_coding;gene_synonym=Bgp3,Cea10 |
| gene19372 | 1.039395755 | 0.03720559 | 67133 | NC_000073.6 | gene_id=gene19372;Dbxref=GeneID:67133,MGI:MGI:1914383;Name=Gp2;description=glycoprotein 2 (zymogen granule membrane);gbkey=Gene;gene=Gp2;gene_biotype=protein_coding;gene_synonym=2310037I18Rik,AV060639 |
| gene38104 | 1.115822109 | 0.03720559 | 57263 | NC_000082.6 | gene_id=gene38104;Dbxref=GeneID:57263,MGI:MGI:1888505;Name=Retnlb;description=resistin like beta;gbkey=Gene;gene=Retnlb;gene_biotype=protein_coding;gene_synonym=9030012B21Rik,Fizz2,Relmb,RELMbeta,Xcp3 |
| gene30846 | 1.106084168 | 0.039933362 | 20716 | NC_000078.6 | gene_id=gene30846;Dbxref=GeneID:20716,MGI:MGI:105045;Name=Serpina3n;description=serine (or cysteine) peptidase inhibitor%2C clade A%2C member 3N;gbkey=Gene;gene=Serpina3n;gene_biotype=protein_coding;gene_synonym=Spi2-2,Spi2.2,Spi2/eb.4 |
| gene14369 | 0.826548162 | 0.043777052 | 93695 | NC_000072.6 | gene_id=gene14369;Dbxref=GeneID:93695,MGI:MGI:1934765;Name=Gpnmb;description=glycoprotein (transmembrane) nmb;gbkey=Gene;gene=Gpnmb;gene_biotype=protein_coding;gene_synonym=DC-HIL,Dchil,ipd |
| gene17785 | 0.98087542 | 0.049001989 | 12489 | NC_000073.6 | gene_id=gene17785;Dbxref=GeneID:12489,MGI:MGI:99440;Name=Cd33;description=CD33 antigen;gbkey=Gene;gene=Cd33;gene_biotype=protein_coding;gene_synonym=gp67,Siglec-3 |
| gene34812 | 1.042877734 | 0.049109706 | 16995 | NC_000080.6 | gene_id=gene34812;Dbxref=GeneID:16995,MGI:MGI:1309472;Name=Ltb4r1;description=leukotriene B4 receptor 1;gbkey=Gene;gene=Ltb4r1;gene_biotype=protein_coding;gene_synonym=BLT1,BLTR,Ltb4r,mBLTR |
| gene37773 | 1.080073171 | 0.049109706 | 16180 | NC_000082.6 | gene_id=gene37773;Dbxref=GeneID:16180,MGI:MGI:104975;Name=Il1rap;description=interleukin 1 receptor accessory protein;gbkey=Gene;gene=Il1rap;gene_biotype=protein_coding;gene_synonym=6430709H04Rik,AI255955,AV239853,IL-1RAcP |
| gene41090 | 1.006293052 | 0.049109706 | 12475 | NC_000084.6 | gene_id=gene41090;Dbxref=GeneID:12475,MGI:MGI:88318;Name=Cd14;description=CD14 antigen;gbkey=Gene;gene=Cd14;gene_biotype=protein_coding |
| gene43127 | 1.079628103 | 0.049592131 | 12979 | NC_000085.6 | gene_id=gene43127;Dbxref=GeneID:12979,MGI:MGI:1339756;Name=Csf1r-ps;gbkey=Gene;gene=Csf1r-ps;gene_biotype=pseudogene;pseudo=true |
| gene21101 | 1.098657914 | 0.050091706 | 72054 | NC_000074.6 | gene_id=gene21101;Dbxref=GeneID:72054,MGI:MGI:1919304;Name=Cyp4f18;description=cytochrome P450%2C family 4%2C subfamily f%2C polypeptide 18;gbkey=Gene;gene=Cyp4f18;gene_biotype=protein_coding;gene_synonym=1810054N16Rik,Cyp4f3,Cypf18 |
