# Supplementary material for: Evidence for the Effect of Vaccination on Host-Pathogen Interactions in a Murine Model of Pulmonary Tuberculosis by Mycobacterium tuberculosis
Source: Front Immunol. 2020 May 19;11:930. doi: 10.3389/fimmu.2020.00930 (PMC7248268; doi:10.3389/fimmu.2020.00930)
Supplement: Supplementary Table 3 — Up-regulated genes in the mice infected by strain 48 compared to those infected by strain 48V. [file Table_3.DOCX]

**Supplementary Table 3.** Up-regulated genes in the mice infected by strain 48 compared to those infected by strain 48V

| **Gene-Id** | **logFold Change** | **p-adjust** | **Gi** | **Locus** | **Description** |
| --- | --- | --- | --- | --- | --- |
| gene2147 | 1.327055135 | 3.17E-08 | 19737 | NC_000067.6 | gene_id=gene2147;Dbxref=GeneID:19737,MGI:MGI:1098434;Name=Rgs5;description=regulator of G-protein signaling 5;gbkey=Gene;gene=Rgs5;gene_biotype=protein_coding;gene_synonym=1110070A02Rik |
| gene10825 | 1.781387236 | 1.47E-07 | 230899 | NC_000070.6 | gene_id=gene10825;Dbxref=GeneID:230899,MGI:MGI:97367;Name=Nppa;description=natriuretic peptide type A;gbkey=Gene;gene=Nppa;gene_biotype=protein_coding;gene_synonym=Anf,ANP,Pnd |
| gene40126 | 1.570639919 | 0.00011546 | 20888 | NC_000083.6 | gene_id=gene40126;Dbxref=GeneID:20888,MGI:MGI:102928;Name=Sult1c1;description=sulfotransferase family%2C cytosolic%2C 1C%2C member 1;gbkey=Gene;gene=Sult1c1;gene_biotype=protein_coding;gene_synonym=(PST)G,mOLFST,P-SULT,ST1C1,Stp2,Sult1a2 |
| gene22252 | 1.118198504 | 0.00015779 | 17392 | NC_000075.6 | gene_id=gene22252;Dbxref=GeneID:17392,MGI:MGI:97010;Name=Mmp3;description=matrix metallopeptidase 3;gbkey=Gene;gene=Mmp3;gene_biotype=protein_coding;gene_synonym=EMS-2,MMP-3,SL-1,SLN-1,SLN1,Stmy1,STR-1,Str1 |
| gene25988 | 0.963709919 | 0.00017693 | 13179 | NC_000076.6 | gene_id=gene25988;Dbxref=GeneID:13179,MGI:MGI:94872;Name=Dcn;description=decorin;gbkey=Gene;gene=Dcn;gene_biotype=protein_coding;gene_synonym=DC,DSPG2,PG40,PGII,PGS2,SLRR1B |
| gene14077 | 1.403690986 | 0.00033826 | 232714 | NC_000072.6 | gene_id=gene14077;Dbxref=GeneID:232714,MGI:MGI:1203495;Name=Mgam;description=maltase-glucoamylase;gbkey=Gene;gene=Mgam;gene_biotype=protein_coding;gene_synonym=6030407P20Rik,MGA |
| gene12293 | 0.895690379 | 0.00046832 | 55985 | NC_000071.6 | gene_id=gene12293;Dbxref=GeneID:55985,MGI:MGI:1888499;Name=Cxcl13;description=chemokine (C-X-C motif) ligand 13;gbkey=Gene;gene=Cxcl13;gene_biotype=protein_coding;gene_synonym=4631412M08Rik,Angie,ANGIE2,BCA-1,BLC,BLR1L,Scyb13 |
| gene32076 | 1.268540274 | 0.00058293 | 20706 | NC_000079.6 | gene_id=gene32076;Dbxref=GeneID:20706,MGI:MGI:894668;Name=Serpinb9b;description=serine (or cysteine) peptidase inhibitor%2C clade B%2C member 9b;gbkey=Gene;gene=Serpinb9b;gene_biotype=protein_coding;gene_synonym=1600019A21Rik,ovalbumin,R86,RP23-391I11.4,SPI-CI,Spi10 |
| gene40470 | 0.951267019 | 0.00082882 | 13078 | NC_000083.6 | gene_id=gene40470;Dbxref=GeneID:13078,MGI:MGI:88590;Name=Cyp1b1;description=cytochrome P450%2C family 1%2C subfamily b%2C polypeptide 1;gbkey=Gene;gene=Cyp1b1;gene_biotype=protein_coding;gene_synonym=CP1B,P4501b1 |
| gene45354 | 1.212216684 | 0.00271102 | 237178 | NC_000086.7 | gene_id=gene45354;Dbxref=GeneID:237178,MGI:MGI:1097157;Name=Ppef1;description=protein phosphatase with EF hand calcium-binding domain 1;gbkey=Gene;gene=Ppef1;gene_biotype=protein_coding;gene_synonym=PPEF-1 |
| gene2233 | 1.184895995 | 0.00342309 | 16429 | NC_000067.6 | gene_id=gene2233;Dbxref=GeneID:16429,MGI:MGI:1333831;Name=Itln1;description=intelectin 1 (galactofuranose binding);gbkey=Gene;gene=Itln1;gene_biotype=protein_coding;gene_synonym=IntL,Itln,Itln2,Itln3,Itln5,Itlna,Lfr |
| gene12859 | 0.950421565 | 0.00416616 | 246728 | NC_000071.6 | gene_id=gene12859;Dbxref=GeneID:246728,MGI:MGI:2180852;Name=Oas2;description=2'-5' oligoadenylate synthetase 2;gbkey=Gene;gene=Oas2;gene_biotype=protein_coding;gene_synonym=Oasl11 |
| gene35066 | 0.930489546 | 0.0044739 | 12759 | NC_000080.6 | gene_id=gene35066;Dbxref=GeneID:12759,MGI:MGI:88423;Name=Clu;description=clusterin;gbkey=Gene;gene=Clu;gene_biotype=protein_coding;gene_synonym=AI893575,ApoJ,Cli,D14Ucla3,Sgp-2,Sgp2,SP-40,Sugp-2 |
| gene8265 | 1.224262538 | 0.00668327 | 12722 | NC_000069.6 | gene_id=gene8265;Dbxref=GeneID:12722,MGI:MGI:1316732;Name=Clca3a1;description=chloride channel accessory 3A1;gbkey=Gene;gene=Clca3a1;gene_biotype=protein_coding;gene_synonym=Cacc,Clca1 |
| gene42601 | 0.654313362 | 0.00695645 | 77125 | NC_000085.6 | gene_id=gene42601;Dbxref=GeneID:77125,MGI:MGI:1924375;Name=Il33;description=interleukin 33;gbkey=Gene;gene=Il33;gene_biotype=protein_coding;gene_synonym=9230117N10Rik,Il-33,Il1f11,NF-HEV |
| gene15115 | 1.236623199 | 0.00757436 | 108169150 | NC_000072.6 | gene_id=gene15115;Dbxref=GeneID:108169150;Name=LOC108169150;gbkey=Gene;gene=LOC108169150;gene_biotype=protein_coding |
| gene17356 | 0.84672878 | 0.00757436 | 233046 | NC_000073.6 | gene_id=gene17356;Dbxref=GeneID:233046,MGI:MGI:2386851;Name=Rasgrp4;description=RAS guanyl releasing protein 4;gbkey=Gene;gene=Rasgrp4;gene_biotype=protein_coding |
| gene45446 | 1.090810947 | 0.00770226 | 17318 | NC_000086.7 | gene_id=gene45446;Dbxref=GeneID:17318,MGI:MGI:1100537;Name=Mid1;description=midline 1;gbkey=Gene;gene=Mid1;gene_biotype=protein_coding;gene_synonym=61B3-R,DXHXS1141,Fxy,Trim18 |
| gene28391 | 1.217266145 | 0.00935795 | 17523 | NC_000077.6 | gene_id=gene28391;Dbxref=GeneID:17523,MGI:MGI:97137;Name=Mpo;description=myeloperoxidase;gbkey=Gene;gene=Mpo;gene_biotype=protein_coding;gene_synonym=mKIAA4033 |
| gene31561 | 0.716673844 | 0.00935795 | 17101 | NC_000079.6 | gene_id=gene31561;Dbxref=GeneID:17101,MGI:MGI:107448;Name=Lyst;description=lysosomal trafficking regulator;gbkey=Gene;gene=Lyst;gene_biotype=protein_coding;gene_synonym=beige,bg,D13Sfk13 |
| gene15117 | 0.828711174 | 0.01025138 | 17119 | NC_000072.6 | gene_id=gene15117;Dbxref=GeneID:17119,MGI:MGI:96908;Name=Mxd1;description=MAX dimerization protein 1;gbkey=Gene;gene=Mxd1;gene_biotype=protein_coding;gene_synonym=AW122478,Mad,Mad1 |
| gene43063 | 1.118653423 | 0.01034976 | 226245 | NC_000085.6 | gene_id=gene43063;Dbxref=GeneID:226245,MGI:MGI:2443041;Name=Plekhs1;description=pleckstrin homology domain containing%2C family S member 1;gbkey=Gene;gene=Plekhs1;gene_biotype=protein_coding;gene_synonym=9930023K05Rik |
| gene36167 | 1.097938395 | 0.01104855 | 15117 | NC_000081.6 | gene_id=gene36167;Dbxref=GeneID:15117,MGI:MGI:107821;Name=Has2;description=hyaluronan synthase 2;gbkey=Gene;gene=Has2;gene_biotype=protein_coding |
| gene9276 | 1.181463988 | 0.01331922 | 18406 | NC_000070.6 | gene_id=gene9276;Dbxref=GeneID:18406,MGI:MGI:97444;Name=Orm2;description=orosomucoid 2;gbkey=Gene;gene=Orm2;gene_biotype=protein_coding;gene_synonym=Agp1,Orm-2 |
| gene14668 | 1.103330864 | 0.01356304 | 243469 | NC_000072.6 | gene_id=gene14668;Dbxref=GeneID:243469,MGI:MGI:96494;Name=Igk;description=immunoglobulin kappa chain complex;gbkey=Gene;gene=Igk;gene_biotype=other;gene_synonym=kappa |
| gene19112 | 0.635398516 | 0.01356304 | 668139 | NC_000073.6 | gene_id=gene19112;Dbxref=GeneID:668139,MGI:MGI:3644223;Name=Gm8995;gbkey=Gene;gene=Gm8995;gene_biotype=misc_RNA |
| gene36138 | 0.795836982 | 0.01356304 | 18383 | NC_000081.6 | gene_id=gene36138;Dbxref=GeneID:18383,MGI:MGI:109587;Name=Tnfrsf11b;description=tumor necrosis factor receptor superfamily%2C member 11b (osteoprotegerin);gbkey=Gene;gene=Tnfrsf11b;gene_biotype=protein_coding;gene_synonym=OCIF,Opg,TR1 |
| gene35064 | 1.083241268 | 0.01379331 | 219151 | NC_000080.6 | gene_id=gene35064;Dbxref=GeneID:219151,MGI:MGI:2444418;Name=Scara3;description=scavenger receptor class A%2C member 3;gbkey=Gene;gene=Scara3;gene_biotype=protein_coding;gene_synonym=APC7,C130058N24Rik,CSR,CSR1,MSLR1,MSRL1 |
| gene24743 | 0.694933571 | 0.01661792 | 22361 | NC_000076.6 | gene_id=gene24743;Dbxref=GeneID:22361,MGI:MGI:108395;Name=Vnn1;description=vanin 1;gbkey=Gene;gene=Vnn1;gene_biotype=protein_coding;gene_synonym=V-1 |
| gene19372 | 1.119170279 | 0.02045189 | 67133 | NC_000073.6 | gene_id=gene19372;Dbxref=GeneID:67133,MGI:MGI:1914383;Name=Gp2;description=glycoprotein 2 (zymogen granule membrane);gbkey=Gene;gene=Gp2;gene_biotype=protein_coding;gene_synonym=2310037I18Rik,AV060639 |
| gene22441 | 1.130537862 | 0.02045189 | 53867 | NC_000075.6 | gene_id=gene22441;Dbxref=GeneID:53867,MGI:MGI:1858212;Name=Col5a3;description=collagen%2C type V%2C alpha 3;gbkey=Gene;gene=Col5a3;gene_biotype=protein_coding |
| gene25580 | 1.133411516 | 0.02206623 | 19152 | NC_000076.6 | gene_id=gene25580;Dbxref=GeneID:19152,MGI:MGI:893580;Name=Prtn3;description=proteinase 3;gbkey=Gene;gene=Prtn3;gene_biotype=protein_coding;gene_synonym=mPR3,PR3 |
| gene34061 | 0.836520141 | 0.02909568 | 78754 | NC_000080.6 | gene_id=gene34061;Dbxref=GeneID:78754,MGI:MGI:1926004;Name=Galnt15;description=UDP-N-acetyl-alpha-D-galactosamine:polypeptide N-acetylgalactosaminyltransferase 15;gbkey=Gene;gene=Galnt15;gene_biotype=protein_coding;gene_synonym=4631401E18Rik,Galntl2,mpp-GalNAc-T15 |
| gene43016 | 1.095982279 | 0.02909568 | 381232 | NC_000085.6 | gene_id=gene43016;Dbxref=GeneID:381232,MGI:MGI:1922001;Name=Mirt1;description=myocardial infarction associated transcript 1;gbkey=Gene;gene=Mirt1;gene_biotype=lncRNA;gene_synonym=4833407H14Rik,5430437C04Rik,5830416P10Rik,ENSMUSG00000074788,Gm10758 |
| gene35168 | 0.851637174 | 0.02924815 | 213053 | NC_000080.6 | gene_id=gene35168;Dbxref=GeneID:213053,MGI:MGI:2384851;Name=Slc39a14;description=solute carrier family 39 (zinc transporter)%2C member 14;gbkey=Gene;gene=Slc39a14;gene_biotype=protein_coding;gene_synonym=FAD-123,fad123,ZIP-14,Zip14 |
| gene19675 | 1.10055924 | 0.02994922 | 12945 | NC_000073.6 | gene_id=gene19675;Dbxref=GeneID:12945,MGI:MGI:106210;Name=Dmbt1;description=deleted in malignant brain tumors 1;gbkey=Gene;gene=Dmbt1;gene_biotype=protein_coding;gene_synonym=CRP,CRP-[a],CRP-[b],Crpd,DBMT1,gp300,p80 |
| gene42995 | 1.07187425 | 0.03929091 | 58178 | NC_000085.6 | gene_id=gene42995;Dbxref=GeneID:58178,MGI:MGI:1929666;Name=Sorcs1;description=sortilin-related VPS10 domain containing receptor 1;gbkey=Gene;gene=Sorcs1;gene_biotype=protein_coding;gene_synonym=mSorCS,Sorcs |
| gene29908 | 1.030824771 | 0.04355426 | 23795 | NC_000078.6 | gene_id=gene29908;Dbxref=GeneID:23795,MGI:MGI:1344405;Name=Agr2;description=anterior gradient 2;gbkey=Gene;gene=Agr2;gene_biotype=protein_coding;gene_synonym=Agr2h,Gob-4,HAG-2,mAG-2,XAG-2 |
| gene427 | 0.676661381 | 0.0448093 | 16177 | NC_000067.6 | gene_id=gene427;Dbxref=GeneID:16177,MGI:MGI:96545;Name=Il1r1;description=interleukin 1 receptor%2C type I;gbkey=Gene;gene=Il1r1;gene_biotype=protein_coding;gene_synonym=CD121a,CD121b,IL-1R1,IL-iR,Il1r-1 |
| gene35303 | 1.027865646 | 0.04650261 | 21943 | NC_000080.6 | gene_id=gene35303;Dbxref=GeneID:21943,MGI:MGI:1100089;Name=Tnfsf11;description=tumor necrosis factor (ligand) superfamily%2C member 11;gbkey=Gene;gene=Tnfsf11;gene_biotype=protein_coding;gene_synonym=Ly109l,ODF,OPG,OPGL,RANKL,Trance |
| gene19832 | 0.991243359 | 0.04790815 | 244233 | NC_000073.6 | gene_id=gene19832;Dbxref=GeneID:244233,MGI:MGI:2443796;Name=Cd163l1;description=CD163 molecule-like 1;gbkey=Gene;gene=Cd163l1;gene_biotype=protein_coding;gene_synonym=B430307C05,E430002D04Rik,Scart1 |
| gene21732 | 0.655242557 | 0.04790815 | 20650 | NC_000074.6 | gene_id=gene21732;Dbxref=GeneID:20650,MGI:MGI:101771;Name=Sntb2;description=syntrophin%2C basic 2;gbkey=Gene;gene=Sntb2;gene_biotype=protein_coding;gene_synonym=Snt2 |
| gene29066 | 0.731441846 | 0.04790815 | 104681 | NC_000077.6 | gene_id=gene29066;Dbxref=GeneID:104681,MGI:MGI:2144585;Name=Slc16a6;description=solute carrier family 16 (monocarboxylic acid transporters)%2C member 6;gbkey=Gene;gene=Slc16a6;gene_biotype=protein_coding;gene_synonym=AW743111,ESTM12,MCT 6,MCT 7,MCT6 |
| gene45041 | 1.042013965 | 0.05008478 | 12738 | NC_000086.7 | gene_id=gene45041;Dbxref=GeneID:12738,MGI:MGI:1276110;Name=Cldn2;description=claudin 2;gbkey=Gene;gene=Cldn2;gene_biotype=protein_coding;gene_synonym=AL022813 |
| gene30665 | 1.017744152 | 0.05009093 | 13371 | NC_000078.6 | gene_id=gene30665;Dbxref=GeneID:13371,MGI:MGI:1338833;Name=Dio2;description=deiodinase%2C iodothyronine%2C type II;gbkey=Gene;gene=Dio2;gene_biotype=protein_coding;gene_synonym=5DII,AI324267,DIOII |
