# Supplementary material for: Evidence for the Effect of Vaccination on Host-Pathogen Interactions in a Murine Model of Pulmonary Tuberculosis by Mycobacterium tuberculosis
Source: Front Immunol. 2020 May 19;11:930. doi: 10.3389/fimmu.2020.00930 (PMC7248268; doi:10.3389/fimmu.2020.00930)
Supplement: Supplementary Table 4 — Down-regulated genes in the mice infected by strain 46 compared to those infected by strain 46P. [file Table_4.DOCX]

**Supplementary Table 4.** Down-regulated genes in the mice infected by strain 46 compared to those infected by strain 46P

| **Gene-Id** | **logFold Change** | **p-adjust** | **Gi** | **Locus** | **Description** |
| --- | --- | --- | --- | --- | --- |
| gene2684 | -1.458064019 | 0.00068714 | 12902 | NC_000067.6 | gene_id=gene2684;Dbxref=GeneID:12902,MGI:MGI:88489;Name=Cr2;description=complement receptor 2;gbkey=Gene;gene=Cr2;gene_biotype=protein_coding;gene_synonym=C3DR,CD21,CD35,Cr-1,Cr-2,Cr1 |
| gene40855 | -1.503257074 | 0.00221544 | 13507 | NC_000084.6 | gene_id=gene40855;Dbxref=GeneID:13507,MGI:MGI:1194993;Name=Dsc3;description=desmocollin 3;gbkey=Gene;gene=Dsc3;gene_biotype=protein_coding;gene_synonym=5430426I24Rik |
| gene30026 | -1.273874499 | 0.01388913 | 12810 | NC_000078.6 | gene_id=gene30026;Dbxref=GeneID:12810,MGI:MGI:1278313;Name=Coch;description=cochlin;gbkey=Gene;gene=Coch;gene_biotype=protein_coding;gene_synonym=AW122937,Coch-5B2,D12H14S564E |
| gene30803 | -1.044243919 | 0.01388913 | 76933 | NC_000078.6 | gene_id=gene30803;Dbxref=GeneID:76933,MGI:MGI:1924183;Name=Ifi27l2a;description=interferon%2C alpha-inducible protein 27 like 2A;gbkey=Gene;gene=Ifi27l2a;gene_biotype=protein_coding;gene_synonym=2310061N23Rik,Ifi27,Isg12,Isg12(b1) |
| gene1564 | -1.065015212 | 0.02671644 | 69169 | NC_000067.6 | gene_id=gene1564;Dbxref=GeneID:69169,MGI:MGI:1916419;Name=Fcmr;description=Fc fragment of IgM receptor;gbkey=Gene;gene=Fcmr;gene_biotype=protein_coding;gene_synonym=1810037B05Rik,Faim3,FcmuR,Toso |
| gene33554 | -0.901741189 | 0.03415356 | 625360 | NC_000079.6 | gene_id=gene33554;Dbxref=GeneID:625360,MGI:MGI:4840510;Name=BC147527;description=cDNA sequence BC147527;gbkey=Gene;gene=BC147527;gene_biotype=protein_coding |
| gene8962 | -0.918228662 | 0.03720559 | 18507 | NC_000070.6 | gene_id=gene8962;Dbxref=GeneID:18507,MGI:MGI:97489;Name=Pax5;description=paired box 5;gbkey=Gene;gene=Pax5;gene_biotype=protein_coding;gene_synonym=BSAP,EBB-1,KLP,Pax-5 |
| gene36520 | -0.907030218 | 0.03804746 | 71898 | NC_000081.6 | gene_id=gene36520;Dbxref=GeneID:71898,MGI:MGI:1919148;Name=Apol9b;description=apolipoprotein L 9b;gbkey=Gene;gene=Apol9b;gene_biotype=protein_coding;gene_synonym=2310016F22Rik |
