# Supplementary material for: Evidence for the Effect of Vaccination on Host-Pathogen Interactions in a Murine Model of Pulmonary Tuberculosis by Mycobacterium tuberculosis
Source: Front Immunol. 2020 May 19;11:930. doi: 10.3389/fimmu.2020.00930 (PMC7248268; doi:10.3389/fimmu.2020.00930)
Supplement: Supplementary Table 5 — Down-regulated genes in the mice infected by strain 48 compared to those infected by strain 48V. [file Table_5.DOCX]

**Supplementary Table 5**. Down-regulated genes in the mice infected by strain 48 compared to those infected by strain 48V

| **Gene-Id** | **logFoldChange** | **padjust** | **Gi** | **Locus** | **Description** |
| --- | --- | --- | --- | --- | --- |
| gene20823 | -1.422979279 | 1.70E-13 | 15446 | NC_000074.6 | gene_id=gene20823;Dbxref=GeneID:15446,MGI:MGI:108085;Name=Hpgd;description=hydroxyprostaglandin dehydrogenase 15 (NAD);gbkey=Gene;gene=Hpgd;gene_biotype=protein_coding;gene_synonym=15-PGDH,AV026552 |
| gene17244 | -1.65392743 | 4.17E-07 | 13107 | NC_000073.6 | gene_id=gene17244;Dbxref=GeneID:13107,MGI:MGI:88608;Name=Cyp2f2;description=cytochrome P450%2C family 2%2C subfamily f%2C polypeptide 2;gbkey=Gene;gene=Cyp2f2;gene_biotype=protein_coding;gene_synonym=Cyp2f |
| gene2572 | -1.370319491 | 1.16E-06 | 16526 | NC_000067.6 | gene_id=gene2572;Dbxref=GeneID:16526,MGI:MGI:109366;Name=Kcnk2;description=potassium channel%2C subfamily K%2C member 2;gbkey=Gene;gene=Kcnk2;gene_biotype=protein_coding;gene_synonym=A430027H14Rik,AI848635,TREK-1 |
| gene23238 | -1.697740318 | 1.71E-05 | 13076 | NC_000075.6 | gene_id=gene23238;Dbxref=GeneID:13076,MGI:MGI:88588;Name=Cyp1a1;description=cytochrome P450%2C family 1%2C subfamily a%2C polypeptide 1;gbkey=Gene;gene=Cyp1a1;gene_biotype=protein_coding;gene_synonym=AHH,AHRR,CP11,Cyp1a2,P450-1 |
| gene43351 | -0.833663389 | 3.00E-05 | 21912 | NC_000086.7 | gene_id=gene43351;Dbxref=GeneID:21912,MGI:MGI:1298407;Name=Tspan7;description=tetraspanin 7;gbkey=Gene;gene=Tspan7;gene_biotype=protein_coding;gene_synonym=1200014P11Rik,A15,AI323365,Cd231,Mxs1,PE31,R74651,TALLA,Tm4sf2 |
| gene852 | -1.52352334 | 0.000111814 | 16008 | NC_000067.6 | gene_id=gene852;Dbxref=GeneID:16008,MGI:MGI:96437;Name=Igfbp2;description=insulin-like growth factor binding protein 2;gbkey=Gene;gene=Igfbp2;gene_biotype=protein_coding;gene_synonym=AI255832,IBP-2,Igfbp-2,mIGFBP-2 |
| gene22051 | -1.214826891 | 0.000145807 | 13479 | NC_000074.6 | gene_id=gene22051;Dbxref=GeneID:13479,MGI:MGI:94917;Name=Dpep1;description=dipeptidase 1 (renal);gbkey=Gene;gene=Dpep1;gene_biotype=protein_coding;gene_synonym=AI327012,MBD |
| gene28434 | -0.977449235 | 0.000145807 | 67888 | NC_000077.6 | gene_id=gene28434;Dbxref=GeneID:67888,MGI:MGI:1915138;Name=Tmem100;description=transmembrane protein 100;gbkey=Gene;gene=Tmem100;gene_biotype=protein_coding;gene_synonym=1810057C19Rik,AV011897 |
| gene14498 | -1.35266694 | 0.000229855 | 21743 | NC_000072.6 | gene_id=gene14498;Dbxref=GeneID:21743,MGI:MGI:102963;Name=Inmt;description=indolethylamine N-methyltransferase;gbkey=Gene;gene=Inmt;gene_biotype=protein_coding;gene_synonym=Temt |
| gene27644 | -0.758206477 | 0.000268147 | 18858 | NC_000077.6 | gene_id=gene27644;Dbxref=GeneID:18858,MGI:MGI:97631;Name=Pmp22;description=peripheral myelin protein 22;gbkey=Gene;gene=Pmp22;gene_biotype=protein_coding;gene_synonym=22kDa,Gas-3,HNPP,Tr,trembler |
| gene30349 | -0.889819362 | 0.000338256 | 238276 | NC_000078.6 | gene_id=gene30349;Dbxref=GeneID:238276,MGI:MGI:2685104;Name=Akap5;description=A kinase (PRKA) anchor protein 5;gbkey=Gene;gene=Akap5;gene_biotype=protein_coding;gene_synonym=3526401B18Rik,AKAP 150,AKAP-5,AKAP150,BB098886,Gm258,P150 |
| gene40580 | -0.732510683 | 0.000582934 | 13819 | NC_000083.6 | gene_id=gene40580;Dbxref=GeneID:13819,MGI:MGI:109169;Name=Epas1;description=endothelial PAS domain protein 1;gbkey=Gene;gene=Epas1;gene_biotype=protein_coding;gene_synonym=bHLHe73,HIF-2alpha,HIF2A,HLF,HRF,MOP2 |
| gene7804 | -1.08793874 | 0.000828823 | 14862 | NC_000069.6 | gene_id=gene7804;Dbxref=GeneID:14862,MGI:MGI:95860;Name=Gstm1;description=glutathione S-transferase%2C mu 1;gbkey=Gene;gene=Gstm1;gene_biotype=protein_coding;gene_synonym=Gstb-1,Gstb1 |
| gene13670 | -1.18367658 | 0.00135267 | 18979 | NC_000072.6 | gene_id=gene13670;Dbxref=GeneID:18979,MGI:MGI:103295;Name=Pon1;description=paraoxonase 1;gbkey=Gene;gene=Pon1;gene_biotype=protein_coding;gene_synonym=Pon |
| gene21974 | -0.799371442 | 0.00135267 | 15227 | NC_000074.6 | gene_id=gene21974;Dbxref=GeneID:15227,MGI:MGI:1347470;Name=Foxf1;description=forkhead box F1;gbkey=Gene;gene=Foxf1;gene_biotype=protein_coding;gene_synonym=AI450827,Foxf1a,Freac-1,FREAC1,HFH-8,Hfh8 |
| gene31134 | -0.710198343 | 0.001732845 | 68337 | NC_000078.6 | gene_id=gene31134;Dbxref=GeneID:68337,MGI:MGI:1915587;Name=Crip2;description=cysteine rich protein 2;gbkey=Gene;gene=Crip2;gene_biotype=protein_coding;gene_synonym=0610010I23Rik,AW743261,C77570,Crp,CRP2,CRP4,ESP1,Hlp |
| gene21619 | -1.342458417 | 0.00262846 | 12556 | NC_000074.6 | gene_id=gene21619;Dbxref=GeneID:12556,MGI:MGI:106671;Name=Cdh16;description=cadherin 16;gbkey=Gene;gene=Cdh16;gene_biotype=protein_coding |
| gene43290 | -1.29119036 | 0.002782157 | 209837 | NC_000086.7 | gene_id=gene43290;Dbxref=GeneID:209837,MGI:MGI:2148066;Name=Slc38a5;description=solute carrier family 38%2C member 5;gbkey=Gene;gene=Slc38a5;gene_biotype=protein_coding;gene_synonym=C81234,E330031E14,JM24,SN2 |
| gene11821 | -1.023974174 | 0.002829965 | 12425 | NC_000071.6 | gene_id=gene11821;Dbxref=GeneID:12425,MGI:MGI:99478;Name=Cckar;description=cholecystokinin A receptor;gbkey=Gene;gene=Cckar;gene_biotype=protein_coding;gene_synonym=AW106902 |
| gene44959 | -1.074685777 | 0.003124559 | 406217 | NC_000086.7 | gene_id=gene44959;Dbxref=GeneID:406217,MGI:MGI:3606746;Name=Bex4;description=brain expressed X-linked 4;gbkey=Gene;gene=Bex4;gene_biotype=protein_coding |
| gene21516 | -1.138567824 | 0.003423086 | 67801 | NC_000074.6 | gene_id=gene21516;Dbxref=GeneID:67801,MGI:MGI:1915051;Name=Pllp;description=plasma membrane proteolipid;gbkey=Gene;gene=Pllp;gene_biotype=protein_coding;gene_synonym=0610010I06Rik,AV001002,Plapi,Tm4sf11 |
| gene43896 | -0.858332434 | 0.004416867 | 66158 | NC_000086.7 | gene_id=gene43896;Dbxref=GeneID:66158,MGI:MGI:1913408;Name=Cxx1a;description=CAAX box 1A;gbkey=Gene;gene=Cxx1a;gene_biotype=protein_coding;gene_synonym=1110012O05Rik,Mar8.2A/B,Mart8b |
| gene37601 | -0.68895805 | 0.005939209 | 12741 | NC_000082.6 | gene_id=gene37601;Dbxref=GeneID:12741,MGI:MGI:1276112;Name=Cldn5;description=claudin 5;gbkey=Gene;gene=Cldn5;gene_biotype=protein_coding;gene_synonym=AI854493,MBEC1,Tmvcf |
| gene13779 | -0.776882921 | 0.006614123 | 12389 | NC_000072.6 | gene_id=gene13779;Dbxref=GeneID:12389,MGI:MGI:102709;Name=Cav1;description=caveolin 1%2C caveolae protein;gbkey=Gene;gene=Cav1;gene_biotype=protein_coding;gene_synonym=Cav,Cav-1 |
| gene44956 | -1.15326395 | 0.006907502 | 12069 | NC_000086.7 | gene_id=gene44956;Dbxref=GeneID:12069,MGI:MGI:1338017;Name=Bex2;description=brain expressed X-linked 2;gbkey=Gene;gene=Bex2;gene_biotype=protein_coding;gene_synonym=AL024066,Bex1 |
| gene19968 | -1.108881879 | 0.007629668 | 16002 | NC_000073.6 | gene_id=gene19968;Dbxref=GeneID:16002,MGI:MGI:96434;Name=Igf2;description=insulin-like growth factor 2;gbkey=Gene;gene=Igf2;gene_biotype=protein_coding;gene_synonym=AL033362,Igf-2,Igf-II,M6pr,Mpr,Peg2 |
| gene27031 | -0.83912621 | 0.008203321 | 216643 | NC_000077.6 | gene_id=gene27031;Dbxref=GeneID:216643,MGI:MGI:2387597;Name=Gabrp;description=gamma-aminobutyric acid (GABA) A receptor%2C pi;gbkey=Gene;gene=Gabrp;gene_biotype=protein_coding |
| gene11674 | -0.725094369 | 0.00935795 | 231162 | NC_000071.6 | gene_id=gene11674;Dbxref=GeneID:231162,MGI:MGI:2684993;Name=Cytl1;description=cytokine-like 1;gbkey=Gene;gene=Cytl1;gene_biotype=protein_coding;gene_synonym=4930443F05Rik,4Cytl1,C17,Cyt1,Gm147 |
| gene37203 | -1.214893373 | 0.009917327 | 223917 | NC_000081.6 | gene_id=gene37203;Dbxref=GeneID:223917,MGI:MGI:2385030;Name=Krt79;description=keratin 79;gbkey=Gene;gene=Krt79;gene_biotype=protein_coding;gene_synonym=BC031593 |
| gene29386 | -1.035057864 | 0.010251381 | 12409 | NC_000077.6 | gene_id=gene29386;Dbxref=GeneID:12409,MGI:MGI:107200;Name=Cbr2;description=carbonyl reductase 2;gbkey=Gene;gene=Cbr2;gene_biotype=protein_coding;gene_synonym=MLCR |
| gene48450 | -0.882307396 | 0.010251381 | 17717 | NC_005089.1 | gene_id=gene48450;Dbxref=GeneID:17717,MGI:MGI:102500;Name=ND2;Note=URF2;gbkey=Gene;gene=ND2;gene_biotype=protein_coding |
| gene43089 | -1.090351284 | 0.010349755 | 14585 | NC_000085.6 | gene_id=gene43089;Dbxref=GeneID:14585,MGI:MGI:1100842;Name=Gfra1;description=glial cell line derived neurotrophic factor family receptor alpha 1;gbkey=Gene;gene=Gfra1;gene_biotype=protein_coding;gene_synonym=AU042498 |
| gene28321 | -0.649452272 | 0.010551399 | 12351 | NC_000077.6 | gene_id=gene28321;Dbxref=GeneID:12351,MGI:MGI:1096574;Name=Car4;description=carbonic anhydrase 4;gbkey=Gene;gene=Car4;gene_biotype=protein_coding;gene_synonym=AW456718,Ca4 |
| gene12779 | -0.964218312 | 0.012194125 | 433943 | NC_000071.6 | gene_id=gene12779;Dbxref=GeneID:433943,MGI:MGI:3648223;Name=Gstm2-ps1;description=glutathione S-transferase mu 2 (muscle)%2C pseudogene 1;gbkey=Gene;gene=Gstm2-ps1;gene_biotype=pseudogene;gene_synonym=EG433943,Gm5562;pseudo=true |
| gene23525 | -0.73188226 | 0.015401118 | 102371 | NC_000075.6 | gene_id=gene23525;Dbxref=GeneID:102371,MGI:MGI:2142908;Name=Myzap;description=myocardial zonula adherens protein;gbkey=Gene;gene=Myzap;gene_biotype=protein_coding;gene_synonym=AA407270,AV006038,Gcom1,Gm640,Grinl1a7,Myozap |
| gene21669 | -0.647604047 | 0.015654694 | 67971 | NC_000074.6 | gene_id=gene21669;Dbxref=GeneID:67971,MGI:MGI:1915221;Name=Tppp3;description=tubulin polymerization-promoting protein family member 3;gbkey=Gene;gene=Tppp3;gene_biotype=protein_coding;gene_synonym=2700055K07Rik,Ceacam9,CGI-38,mmCGM8 |
| gene7020 | -0.894463569 | 0.015654694 | 68659 | NC_000069.6 | gene_id=gene7020;Dbxref=GeneID:68659,MGI:MGI:1915909;Name=Fam198b;description=family with sequence similarity 198%2C member B;gbkey=Gene;gene=Fam198b;gene_biotype=protein_coding;gene_synonym=1110032E23Rik,2210419I08Rik,AV011458,Ened |
| gene14486 | -0.870347755 | 0.018410756 | 69938 | NC_000072.6 | gene_id=gene14486;Dbxref=GeneID:69938,MGI:MGI:1917188;Name=Scrn1;description=secernin 1;gbkey=Gene;gene=Scrn1;gene_biotype=protein_coding;gene_synonym=2810019K23Rik,6330535A03Rik,AI852905,mKIAA0193,SES1 |
| gene34260 | -1.010163281 | 0.018410756 | 13587 | NC_000080.6 | gene_id=gene34260;Dbxref=GeneID:13587,MGI:MGI:108020;Name=Ear2;description=eosinophil-associated%2C ribonuclease A family%2C member 2;gbkey=Gene;gene=Ear2;gene_biotype=protein_coding;gene_synonym=EAR-13,EAR-2,ECP,ECP 2,Raf3,Rnase2 |
| gene37352 | -0.686135444 | 0.018410756 | 239691 | NC_000082.6 | gene_id=gene37352;Dbxref=GeneID:239691,MGI:MGI:2146559;Name=AU021092;description=expressed sequence AU021092;gbkey=Gene;gene=AU021092;gene_biotype=protein_coding |
| gene41259 | -0.904321125 | 0.020451893 | 117158 | NC_000084.6 | gene_id=gene41259;Dbxref=GeneID:117158,MGI:MGI:2153470;Name=Scgb3a2;description=secretoglobin%2C family 3A%2C member 2;gbkey=Gene;gene=Scgb3a2;gene_biotype=protein_coding;gene_synonym=LuLeu1,Pnsp1,UGRP1,Utgrp1 |
| gene30130 | -0.595154655 | 0.020622482 | 66864 | NC_000078.6 | gene_id=gene30130;Dbxref=GeneID:66864,MGI:MGI:1914114;Name=Clec14a;description=C-type lectin domain family 14%2C member a;gbkey=Gene;gene=Clec14a;gene_biotype=protein_coding;gene_synonym=1200003C23Rik,AI642649 |
| gene43962 | -0.583451502 | 0.022066229 | 14199 | NC_000086.7 | gene_id=gene43962;Dbxref=GeneID:14199,MGI:MGI:1298387;Name=Fhl1;description=four and a half LIM domains 1;gbkey=Gene;gene=Fhl1;gene_biotype=protein_coding;gene_synonym=FHL-1,KyoT,RAM14-1,SLIM,SLIM-1 |
| gene19986 | -0.762236834 | 0.023423984 | 12577 | NC_000073.6 | gene_id=gene19986;Dbxref=GeneID:12577,MGI:MGI:104564;Name=Cdkn1c;description=cyclin-dependent kinase inhibitor 1C (P57);gbkey=Gene;gene=Cdkn1c;gene_biotype=protein_coding;gene_synonym=AL024410,CDKI,Kip2,p57(kip2),p57Kip2 |
| gene43897 | -0.701471567 | 0.025044034 | 553127 | NC_000086.7 | gene_id=gene43897;Dbxref=GeneID:553127,MGI:MGI:3576504;Name=Cxx1b;description=CAAX box 1B;gbkey=Gene;gene=Cxx1b;gene_biotype=protein_coding;gene_synonym=Mart8c |
| gene899 | -1.090275207 | 0.025044034 | 241113 | NC_000067.6 | gene_id=gene899;Dbxref=GeneID:241113,MGI:MGI:1891343;Name=Prkag3;description=protein kinase%2C AMP-activated%2C gamma 3 non-catatlytic subunit;gbkey=Gene;gene=Prkag3;gene_biotype=protein_coding;gene_synonym=Amkg3,Ampkg3,AMPKg3L,AMPKg3S |
| gene28086 | -1.035726499 | 0.02728479 | 15567 | NC_000077.6 | gene_id=gene28086;Dbxref=GeneID:15567,MGI:MGI:96285;Name=Slc6a4;description=solute carrier family 6 (neurotransmitter transporter%2C serotonin)%2C member 4;gbkey=Gene;gene=Slc6a4;gene_biotype=protein_coding;gene_synonym=5-HTT,AI323329,Htt,Sert |
| gene32179 | -0.947782603 | 0.02728479 | 12161 | NC_000079.6 | gene_id=gene32179;Dbxref=GeneID:12161,MGI:MGI:88182;Name=Bmp6;description=bone morphogenetic protein 6;gbkey=Gene;gene=Bmp6;gene_biotype=protein_coding;gene_synonym=D13Wsu115e,Vgr1 |
| gene36125 | -1.044947192 | 0.028735777 | 239435 | NC_000081.6 | gene_id=gene36125;Dbxref=GeneID:239435,MGI:MGI:2181621;Name=Aard;description=alanine and arginine rich domain containing protein;gbkey=Gene;gene=Aard;gene_biotype=protein_coding;gene_synonym=A5D3,AV328152 |
| gene21477 | -0.691986822 | 0.029095678 | 104158 | NC_000074.6 | gene_id=gene21477;Dbxref=GeneID:104158,MGI:MGI:2148202;Name=Ces1d;description=carboxylesterase 1D;gbkey=Gene;gene=Ces1d;gene_biotype=protein_coding;gene_synonym=Ces3,TGH |
| gene26616 | -0.839717705 | 0.031282734 | 380683 | NC_000077.6 | gene_id=gene26616;Dbxref=GeneID:380683,MGI:MGI:3617848;Name=Sec14l3;description=SEC14-like lipid binding 3;gbkey=Gene;gene=Sec14l3;gene_biotype=protein_coding;gene_synonym=1110069O07Rik |
| gene28814 | -0.555382124 | 0.031384003 | 54409 | NC_000077.6 | gene_id=gene28814;Dbxref=GeneID:54409,MGI:MGI:1859650;Name=Ramp2;description=receptor (calcitonin) activity modifying protein 2;gbkey=Gene;gene=Ramp2;gene_biotype=protein_coding |
| gene21279 | -1.025353823 | 0.035554886 | 70134 | NC_000074.6 | gene_id=gene21279;Dbxref=GeneID:70134,MGI:MGI:1917384;Name=2210011C24Rik;description=RIKEN cDNA 2210011C24 gene;gbkey=Gene;gene=2210011C24Rik;gene_biotype=protein_coding |
| gene26830 | -0.853520586 | 0.035554886 | 55963 | NC_000077.6 | gene_id=gene26830;Dbxref=GeneID:55963,MGI:MGI:2135601;Name=Slc1a4;description=solute carrier family 1 (glutamate/neutral amino acid transporter)%2C member 4;gbkey=Gene;gene=Slc1a4;gene_biotype=protein_coding;gene_synonym=ASCT-1,ASCT1,AW045657,SATT |
| gene42473 | -0.64900839 | 0.035554886 | 11668 | NC_000085.6 | gene_id=gene42473;Dbxref=GeneID:11668,MGI:MGI:1353450;Name=Aldh1a1;description=aldehyde dehydrogenase family 1%2C subfamily A1;gbkey=Gene;gene=Aldh1a1;gene_biotype=protein_coding;gene_synonym=Ahd-2,Ahd2,Aldh1,Aldh1a2,E1,Raldh1 |
| gene22704 | -0.809039756 | 0.040845368 | 64011 | NC_000075.6 | gene_id=gene22704;Dbxref=GeneID:64011,MGI:MGI:1927184;Name=Nrgn;description=neurogranin;gbkey=Gene;gene=Nrgn;gene_biotype=protein_coding;gene_synonym=0710001B06Rik,AI838505,NG,NG/RC3,Pss1,R75334,RC3 |
| gene39913 | -0.604538989 | 0.042917358 | 224792 | NC_000083.6 | gene_id=gene39913;Dbxref=GeneID:224792,MGI:MGI:2182928;Name=Adgrf5;description=adhesion G protein-coupled receptor F5;gbkey=Gene;gene=Adgrf5;gene_biotype=protein_coding;gene_synonym=8430401C09Rik,9330185D23,AI528491,Gpr116,mKIAA0758 |
| gene15487 | -0.876752092 | 0.044378886 | 20965 | NC_000072.6 | gene_id=gene15487;Dbxref=GeneID:20965,MGI:MGI:103020;Name=Syn2;description=synapsin II;gbkey=Gene;gene=Syn2;gene_biotype=protein_coding;gene_synonym=2900074L19Rik,AI836018,AI841723 |
| gene18757 | -0.59464162 | 0.046059236 | 17760 | NC_000073.6 | gene_id=gene18757;Dbxref=GeneID:17760,MGI:MGI:1201690;Name=Map6;description=microtubule-associated protein 6;gbkey=Gene;gene=Map6;gene_biotype=protein_coding;gene_synonym=2810411E12Rik,Map-6,Mtap6,STOP |
| gene2038 | -0.932690524 | 0.047908146 | 14262 | NC_000067.6 | gene_id=gene2038;Dbxref=GeneID:14262,MGI:MGI:1100496;Name=Fmo3;description=flavin containing monooxygenase 3;gbkey=Gene;gene=Fmo3;gene_biotype=protein_coding;gene_synonym=AW111792 |
| gene17367 | -0.749586049 | 0.050084784 | 68458 | NC_000073.6 | gene_id=gene17367;Dbxref=GeneID:68458,MGI:MGI:1931139;Name=Ppp1r14a;description=protein phosphatase 1%2C regulatory (inhibitor) subunit 14A;gbkey=Gene;gene=Ppp1r14a;gene_biotype=protein_coding;gene_synonym=1110001M11Rik,Cpi17 |
| gene20194 | -0.554509178 | 0.050084784 | 14068 | NC_000074.6 | gene_id=gene20194;Dbxref=GeneID:14068,MGI:MGI:109325;Name=F7;description=coagulation factor VII;gbkey=Gene;gene=F7;gene_biotype=protein_coding;gene_synonym=AI132620,Cf7,FVII |
| gene26392 | -0.730257723 | 0.050084784 | 407790 | NC_000076.6 | gene_id=gene26392;Dbxref=GeneID:407790,MGI:MGI:3039567;Name=Ndufa4l2;description=NADH dehydrogenase (ubiquinone) 1 alpha subcomplex%2C 4-like 2;gbkey=Gene;gene=Ndufa4l2;gene_biotype=protein_coding;gene_synonym=BC064011 |
| gene40075 | -0.769669309 | 0.050084784 | 76441 | NC_000083.6 | gene_id=gene40075;Dbxref=GeneID:76441,MGI:MGI:1923691;Name=Daam2;description=dishevelled associated activator of morphogenesis 2;gbkey=Gene;gene=Daam2;gene_biotype=protein_coding;gene_synonym=2310016D11Rik,AI843643,AW557870 |
| gene41304 | -0.603270438 | 0.050084784 | 12583 | NC_000084.6 | gene_id=gene41304;Dbxref=GeneID:12583,MGI:MGI:105925;Name=Cdo1;description=cysteine dioxygenase 1%2C cytosolic;gbkey=Gene;gene=Cdo1;gene_biotype=protein_coding;gene_synonym=1300002L19Rik,Cdo,D18Ucla3 |
| gene15720 | -0.63676083 | 0.050090925 | 69202 | NC_000072.6 | gene_id=gene15720;Dbxref=GeneID:69202,MGI:MGI:1916452;Name=Ptms;description=parathymosin;gbkey=Gene;gene=Ptms;gene_biotype=protein_coding;gene_synonym=2610009E16Rik |
| gene23851 | -0.993732542 | 0.050200288 | 76477 | NC_000075.6 | gene_id=gene23851;Dbxref=GeneID:76477,MGI:MGI:1923727;Name=Pcolce2;description=procollagen C-endopeptidase enhancer 2;gbkey=Gene;gene=Pcolce2;gene_biotype=protein_coding;gene_synonym=2400001O18Rik,Pcpe2 |
| gene6920 | -0.910506108 | 0.050200288 | 109222 | NC_000069.6 | gene_id=gene6920;Dbxref=GeneID:109222,MGI:MGI:1924461;Name=Rarres1;description=retinoic acid receptor responder (tazarotene induced) 1;gbkey=Gene;gene=Rarres1;gene_biotype=protein_coding;gene_synonym=5430417P09Rik,AI662122 |
| gene27838 | -0.708119943 | 0.050707385 | 216867 | NC_000077.6 | gene_id=gene27838;Dbxref=GeneID:216867,MGI:MGI:2663709;Name=Slc16a11;description=solute carrier family 16 (monocarboxylic acid transporters)%2C member 11;gbkey=Gene;gene=Slc16a11;gene_biotype=protein_coding;gene_synonym=AB041591,MCT 11,MNCb-2717 |
